# Trends in the incidence of childhood type 1 diabetes after the COVID-19 lockdown in Finland: An observational study

**DOI:** 10.1371/journal.pmed.1005288

**Published:** 2026-11-02

**Authors:** Susanna Tall, Maaret Turtinen, Anna Parviainen, Jenni Pettersson, Anna But, Taina Härkönen, Jussi Hepojoki, Tarja Sironen, Rommel Iheozor-Ejiofor, Hasan Uğurlu, Kalle Saksela, Jorma Ilonen, Suvi M. Virtanen, Olli Vapalahti, Mikael Knip, on behalf of the Finnish Pediatric Diabetes Register

**Affiliations:** 1 Research Program for Clinical and Molecular Metabolism, Faculty of Medicine, University of Helsinki, Helsinki, Finland; 2 Department of Public Health, Finnish Institute for Health and Welfare, Helsinki, Finland; 3 Pediatric Research Center, New Children’s Hospital, Helsinki University Hospital, Helsinki, Finland; 4 Department of Virology, Medicum, University of Helsinki, Helsinki, Finland; 5 Department of Veterinary Biosciences, University of Helsinki, Helsinki, Finland; 6 Biostatistics Consulting, Department of Public Health, Helsinki University Hospital, University of Helsinki, Helsinki, Finland; 7 Erzurum Technical University, Erzurum, Turkey; 8 Virology and Immunology, Helsinki University Hospital Diagnostic Center, Helsinki, Finland; 9 Immunogenetics Laboratory, Institute of Biomedicine, University of Turku, Turku, Finland; 10 Tampere Center for Child, Adolescent and Maternal Health Research, Tampere University and Tampere University Hospital, Tampere, Finland; 11 Tampere University Hospital, Wellbeing Services County of Pirkanmaa, Tampere, Finland; 12 Faculty of Social Sciences, Unit of Health Sciences, Tampere University, Tampere, Finland; 13 Turku Bioscience Centre, University of Turku and Åbo Akademi University, Turku, Finland; Shanghai Jiao Tong University Affiliated Sixth People’s Hospital, CHINA

## Abstract

**Background:**

While COVID-19 lockdowns have been systematically associated with increased incidence rate of type 1 diabetes, less is known about the disease rates after lockdowns. We compared the incidence of type 1 diabetes after the COVID-19 pandemic-related closure of society (i) to the time when lockdown was implemented and to (ii) the time before the pandemic in Finland.

**Methods and findings:**

The incidence of type 1 diabetes after the lockdown (09/2021–12/2022) was compared to the incidence during the lockdown (03/2020–08/2021) and before the lockdown (three 18-month terms 2014–2019) in Finnish children under the age of 15 years. Previous exposure to SARS-CoV-2 was identified by combining information on COVID-19 vaccination with data on SARS-CoV-2 antibodies analyzed in blood samples. The temporal cohort included 3,467 pediatric type 1 diabetes cases, distributed across post-lockdown (*n* = 586), lockdown (*n* = 785), and pre-pandemic (*n* = 2,096) periods. The incidence of type 1 diabetes after the lockdown was significantly lower than the incidence during the lockdown [incidence rate ratio (IRR = 0.85; 95% Cl [0.77,0.95]; *p =* 0.004)]. The rate of type 1 diabetes after the lockdown did not significantly differ from the time before lockdown (IRR = 1.00; 95% Cl [0.91,1.09]; *p* = 0.932). The frequency of children testing positive for infection-associated SARS-CoV-2 antibodies increased from 0.9% during the lockdown to more than 40% after the lockdown. Study limitations include a relatively short follow-up period after the lockdown and the lack of explicit modeling of the underlying long-term secular trend in type 1 diabetes incidence.

**Conclusions:**

The lockdown represents a peak in childhood type 1 diabetes incidence in Finland compared to both the pre-pandemic and post-lockdown periods. However, the reversion toward historical levels after the lockdown does not necessarily establish a return to the expected historical trajectory, nor can it fully distinguish a transient change from temporal compensation superimposed on the background secular trend. The contrasting patterns between type 1 diabetes incidence and SARS-CoV-2 antibody prevalence during and after the lockdown suggest an indirect rather than a direct role of the virus in triggering the observed changes in incidence.

## Author summary

**Why was this study done?:**

- The Coronavirus disease 2019 (COVID-19) pandemic has been linked to an increased incidence of type 1 diabetes in numerous research articles.
- A few European studies have reported that the surge in type 1 diabetes incidence observed during the 2021 lockdowns did not persist after the reopening of society in 2022. However, studies investigating type 1 diabetes incidence beyond the acute phase of the pandemic remain scarce.

**What did the researchers do and find?:**

- The incidence of type 1 diabetes after the lockdown (09/2021–12/2022) was compared to the incidence during (03/2020–08/2021) and before lockdown (three 18-month terms 2014–2019) in Finnish children under the age of 15 years.
- The incidence of type 1 diabetes decreased by 15% among Finnish children during the first 16 months after the COVID-19 lockdown compared with the time when lockdown was implemented.
- The incidence after the lockdown was equal compared to the reference time before the COVID-19 pandemic.

**What do these findings mean?:**

- The lockdown represents a peak in type 1 diabetes incidence in Finland, not only compared to the time before lockdown, but also afterwards.
- Study limitations include a relatively short follow-up period after the lockdown and the lack of explicit modeling of the underlying long-term secular trend in type 1 diabetes incidence.

## Abstract 3

In this observational study, Susanna Tall and colleagues found that childhood type 1 diabetes incidence declined in Finland after COVID-19 lockdown restrictions ended, returning to levels comparable with those observed before the pandemic. Their findings suggest that the elevated incidence seen during the lockdown period represented a temporary peak.

## Introduction

The Coronavirus disease 2019 (COVID-19) pandemic has been related to an increased incidence of type 1 diabetes in a series of research articles [1]. A meta-analysis published in 2023 including 17 studies showed a higher incidence rate of type 1 diabetes during the first year of the pandemic compared with the pre-pandemic period [1]. A few European studies have reported that the 2021 peak in type 1 diabetes incidence did not persist into 2022 [2,3]. Type 1 diabetes incidence in Sweden peaked in 2021 during a period of internationally unique, minimal COVID-19 pandemic-related social distancing restrictions, followed by a slowing rate in 2022 and a return to pre-pandemic trends in 2023 [4]. However, studies investigating the incidence of type 1 diabetes beyond the acute phase of the pandemic remain scarce.

Several meta-analyses have suggested that the severe acute respiratory syndrome coronavirus 2 (SARS-CoV-2) might directly cause type 1 diabetes [5]^,^ whereas some reports indicate that currently there is no convincing evidence to support this view. Notably, only a small proportion of published pandemic-era articles have pursued biochemical confirmation of autoimmunity of the newly diagnosed diabetes cases via systematic islet autoantibody measurements [5]. Consequently, some type 2 diabetes cases may have been misclassified as type 1 diabetes across studies, thereby falsely adding to the observed type 1 diabetes peaks in different countries.

Less than 1% of Finnish children diagnosed with type 1 diabetes during the first 18 months of the pandemic tested positive for infection-induced SARS-CoV-2 antibodies (Figs 1 and S1) [6]. This finding is in line with reports from the USA and Denmark showing that the prevalence of SARS-CoV-2 infections was similar during lockdown in patients with type 1 diabetes to that seen in the population unaffected by type 1 diabetes [7,8]. However, in young children with strong genetic risk for type 1 diabetes, SARS-CoV-2 infection has been associated with the development of islet autoantibodies in two European studies [9,10]. Within the German Fr1da study, in a sub-cohort of 509 children with positivity to multiple islet autoantibodies, the rate of progression to clinical type 1 diabetes after COVID-19 infection was significantly accelerated when compared with individuals who had not been diagnosed with COVID-19 [11].

**Fig 1:**
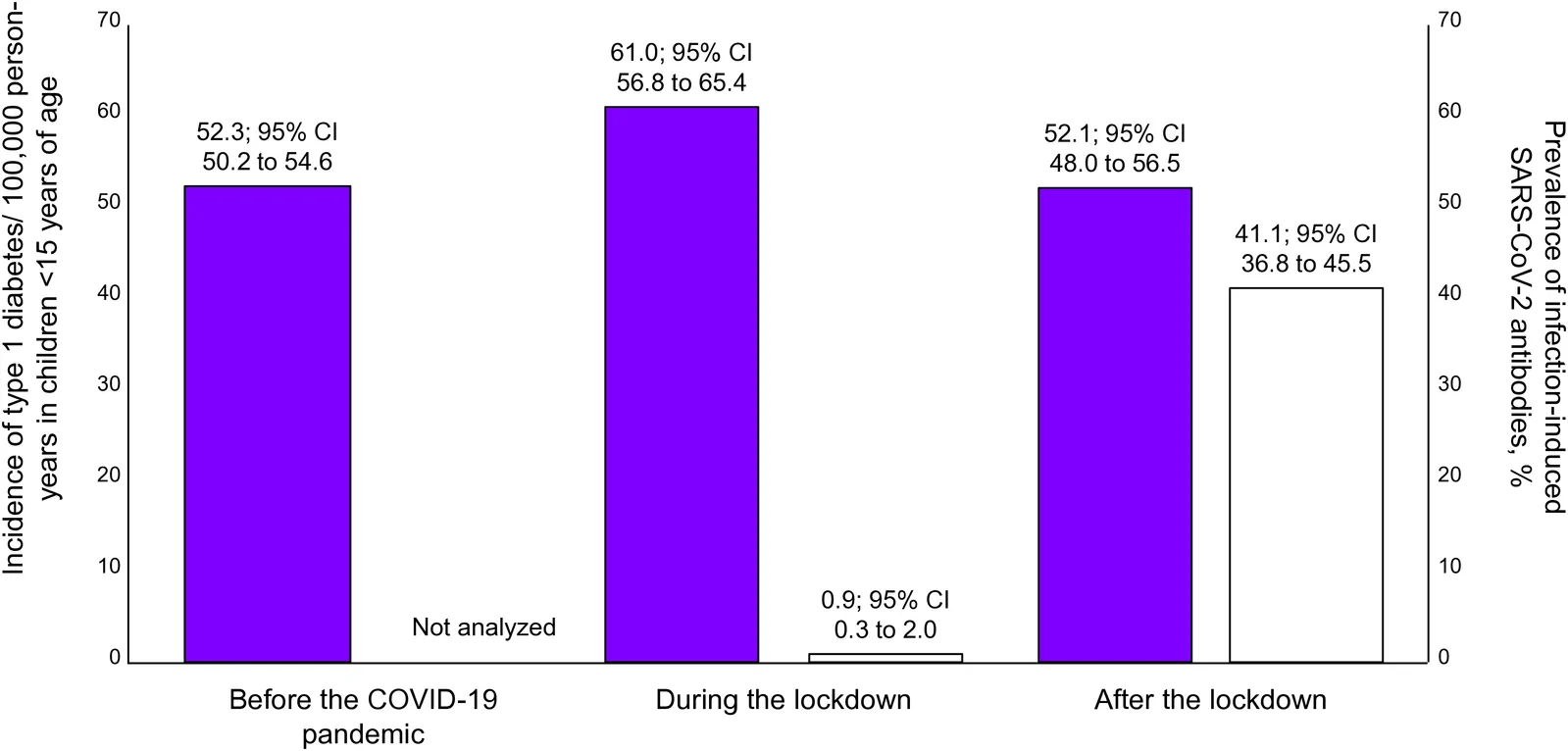
Incidence rates of type 1 diabetes and prevalence of infection-induced SARS-CoV-2 antibodies across study periods. The graph displays data stratified by the reference period before the COVID-19 pandemic (three 18-month blocks spanning 2014–2019), during the societal lockdown (March 2020–August 2021), and after the lockdown (September 2021–December 2022). Blue bars represent the incidence rate of type 1 diabetes (expressed as new cases per 100,000 person-years in children aged <15 years) corresponding to the left y-axis. White bars represent the prevalence (%) of infection-induced SARS-CoV-2 antibodies among children newly diagnosed with type 1 diabetes corresponding to the right y-axis. Exact estimates and 95% confidence intervals (CIs) are shown above each bar. SARS-CoV-2 antibody status was not analyzed for the pre-pandemic reference cohort.

Our primary aim was to compare the incidence of type 1 diabetes after the COVID-19 pandemic-related closure of the society (i) to the time when lockdown was implemented and to (ii) the time before the pandemic in Finland. Previous exposure to SARS-CoV-2 was identified by combining information on COVID-19 vaccination with data on SARS-CoV-2 antibodies analyzed in blood samples. We also compared the clinical characteristics, metabolic decompensation at diagnosis, signs of islet autoimmunity, and frequency of risk-associated human leukocyte antigen (HLA) genotypes in children diagnosed with type 1 diabetes during the pandemic and the reference periods. Based on other European studies [2,3], we expected that the lockdown measures would be associated with a higher incidence of type 1 diabetes. Consequently, we hypothesized that the disease rates after the lockdown would return to levels close to those observed before the lockdown started in Finland.

## Methods

We used data from the Finnish Pediatric Diabetes Register (FPDR) that covers more than 90% of Finnish children with newly diagnosed diabetes [12,13]. The data includes information on diabetes-associated autoantibodies and HLA genotypes. Approximately 99% of the participants have type 1 diabetes, diagnosed based on clinical characteristics, autoantibody status, and HLA genotype, and more than 98% are of European ancestry. The incidence of type 1 diabetes after the lockdown (09/2021–12/2022) was compared to the incidence during (03/2020–08/2021) and before lockdown (three 18-month terms 2014–2019) in Finnish children under the age of 15 years. A detailed description of lockdown measures applied in Finland is provided in the S2 Text.

### Analyses of clinical variables, autoantibodies and HLA genotypes

To determine metabolic decompensation, blood pH and concentrations of plasma β-hydroxybutyrate, glucose, and hemoglobin A_1c_ (HbA1c) were analyzed from blood samples collected from children with newly diagnosed type 1 diabetes. A physician assessed the level of consciousness (normal, altered, and unconscious) for each subject at the time of type 1 diabetes diagnosis. The duration of symptoms associated with type 1 diabetes before diagnosis was assessed through a questionnaire given to the family of each patient. Diabetic ketoacidosis (DKA) was defined as a blood pH < 7.30 in combination with ketonemia. Severe DKA was defined as a blood pH < 7.10.

More than 80% of the children with type 1 diabetes in the FPDR provided blood samples, which were taken at a median of 6 days after the diagnosis for the isolation of serum and peripheral blood mononuclear cells. Insulin autoantibodies (IAA) and autoantibodies to glutamic acid decarboxylase (GADA), islet cell antigen-2 (IA-2A) as well as zinc transporter antigen 8 (ZnT8A) were analyzed from the serum samples with specific radiobinding assays [14] as described in the S3 Text. Serum samples collected more than 14 days after the start of insulin treatment were not included in the insulin autoantibody analyses. An EDTA sample was collected and used for HLA genotyping. Typing of the major HLA-DR-DQ haplotypes was done with PCR-based lanthanide-labeled hybridization and time-resolved fluorometry detection [15]. HLA class II–conferred risk for type 1 diabetes was assessed by classifying the study participants based on their HLA genotypes into six risk groups, ranging from a strongly reduced risk to a high risk [16].

A formal, written consent was obtained from the parent/guardian/head of household of the participating children. The study protocol of the FPDR was approved by the Ethics Committee of the Hospital District of Helsinki and Uusimaa (Helsinki, Finland) (Approval number: 454/E7/2001) (S1 Protocol).

### Analyses of SARS-CoV-2 antibodies

Serum samples taken on a median of 6 days after the diagnosis of type 1 diabetes were analyzed for SARS-CoV-2 antibodies. Samples were first screened with an enzyme-linked immunosorbent assay (ELISA) for IgG antibodies against the SARS-CoV-2 spike protein (S) using a 384-well-based high-throughput ELISA [17]. The positive samples were further titrated for neutralizing antibodies to contemporary variants and those used in vaccines for children in Finland using a pseudovirus neutralization assay based on luciferase-encoded lentiviruses [17,18]. Detailed description of SARS-CoV-2 analyses is provided in the S4 Text.

COVID-19 vaccinations started in Finnish children in late summer 2021. We obtained vaccination data for all type 1 diabetes cases diagnosed after the lockdown (09/2021–12/2022) study period from the Finnish Vaccination Register. To estimate the proportion of children diagnosed after the lockdown who had an antibody profile compatible with previous infection, we applied two distinct criteria: strict and loose (definitions for these criteria are provided in S1 Table).

### Statistical analyses

Statistical analyses were carried out using SAS 9.4, SAS Enterprise Guide for Windows version 7.1 and R Software for Statistical Computing for Windows, versions 4.4.1. A two-tailed *P* value of  .05 or less was considered significant. We calculated overall, sex-specific, and age-specific incidence rates of type 1 diabetes per 100,000 person-years (PY) during the time before, during and after the lockdown. We separated the study population into three age categories: <5 years, 5 to <10 years, and 10 to <15 years. Annual population statistics obtained from Statistics Finland were used to determine person-years in each of the age and sex groups. The type 1 diabetes incidence rate ratios (IRR) and their 95% confidence intervals (CIs) were assessed by fitting multiplicative Poisson regression models on counts of type 1 diabetes cases aggregated by age groups, sex and pandemic timeline, using the natural logarithm of the corresponding PY as an offset term. We applied robust standard errors calculated using the sandwich method (the Huber-White estimator). As a sensitivity analysis, the incidence after the lockdown was compared to the most recent preceding 18-month period before the lockdown (March 1, 2018 to August 31, 2019).

Differences in clinical, metabolic, genetic and immunological characteristics were assessed between the study cohorts. Age at diagnosis and plasma glucose were analyzed with linear regression analysis. HbA_1c_%, HbA_1c_ mmol/mol, blood pH, plasma β-hydroxybutyrate, and all autoantibodies measured as relative units (RU) were analyzed with quantile regression analysis for median. Dichotomous response variables were analyzed with logistic regression analysis. Duration of symptoms and number of family members with type 1 diabetes were analyzed using multinomial logistic regression analysis. We considered sex and age at diagnosis as confounders and controlled for their effect through adjustment in all analyses when appropriate (unadjusted results are provided in S2 and S3 Tables).

### Use of AI disclosure

A Large Language Model (LLM) assistant (Google Gemini, September 2026 version) was utilized during the revision stage of this manuscript. The tool was used exclusively for language editing, text polishing, and improving the stylistic flow and conciseness of the narrative text. Every suggestion provided by the AI tool was thoroughly evaluated, critically reviewed, and verified by the authors to ensure scientific accuracy and contextual alignment with the study. The usage of this tool was restricted strictly to text editing; no study concepts, primary hypotheses, raw datasets, statistical models, figure data, or supporting materials were generated, altered, or affected by the AI tool. The authors retain sole and absolute responsibility for the content, integrity, and final outputs of the manuscript.

## Results

### Incidence rates and incidence rate ratios

Overall, 586 children were diagnosed with type 1 diabetes after the lockdown and 785 during the lockdown, the corresponding incidence rates being 52.1 (95% confidence interval [CI] [48.0,56.5]) and 61.0 (95% CI [56.8,65.4]), respectively (Fig 1). The IRR was 0.85 (95% Cl [0.77,0.95]; *p =* 0*.*004) when both sexes were analyzed together (Fig 2). We found evidence of a reduced type 1 diabetes incidence in males (IRR = 0.82, 95% Cl [0.72,0.95]; *p =* 0*.*007) but the decline in the incidence in females did not reach significance (IRR = 0.90, 95% Cl [0.76,1.05]; *p =* 0.185) (Fig 2). The incidence of type 1 diabetes after the lockdown was similar to that observed before the COVID-19 pandemic (IRR = 1.00, 95% CI [0.91,1.09]; IR = 52.3, 95% CI [50.2,54.6]; *n* = 2,096, *p* = 0.932). Further analyses for different age groups did not provide evidence for any additional differences between cohorts (Fig 2 and Table 1). In the sensitivity analysis, the incidence of type 1 diabetes was 51.2 per 100,000 person-years (95% CI [47.4,55.2]) in the most recent preceding 18-month period before the lockdown being close to that observed during the post-lockdown time (IRR = 1.04, 95% CI [0.91,1.20]; *p* = 0.555).

**Table 1:** Incidence rates of type 1 diabetes in Finnish children under the age of 15 years per 100,000 person-years before (three 18-month terms 2014–2019), during (03/2020–08/2021) and after (09/2021–12/2022) the lockdown.

	Before lockdown (*n* = 2,096)	During lockdown (*n* = 785)	After lockdown (*n* = 586)
All	52.3 (50.1,54.8)	61.0 (56.8,65.4)	52.1 (48.0,56.5)
Females	46.5 (43.5,49.6)	52.0 (46.5,56.0)	46.6 (41.0,52.6)
Males	57.8 (54.6,61.2)	69.6 (63.4,76.3)	57.4 (51.4,63.9)
Age <5	39.6 (36.2,43.2)	47.0 (40.2,54.5)	37.2 (30.9,44.6)
Age <5, females	38.7 (34.0,44.0)	40.0 (31.3,50.3)	34.6 (26.0,45.1)
Age <5, males	40.4 (35.6,45.6)	53.6 (43.7,65.2)	39.8 (30.7,50.7)
Age 5 to <10	59.3 (55.3,63.5)	70.0 (62.4,78.2)	62.0 (54.3,70.3)
Age 5 to <10, females	52.8 (47.5,58.5)	62.2 (52.2,73.6)	59.6 (49.1,71.8)
Age 5 to <10, males	65.6 (59.7,71.8)	77.4 (66.4,89.6)	64.2 (53.5,76.4)
Age 10 to <15	57.0 (53.1,61.2)	63.5 (56.5,71.1)	54.4 (47.6,61.9)
Age 10 to <15, females	47.3 (42.2,52.9)	51.7 (42.8,61.9)	43.7 (35.1,53.7)
Age 10 to <15, males	66.3 (60.3,72.6)	74.8 (64.2,86.5)	64.6 (54.3,76.4)

Data shown as rate (95% Cl).

**Fig 2:**
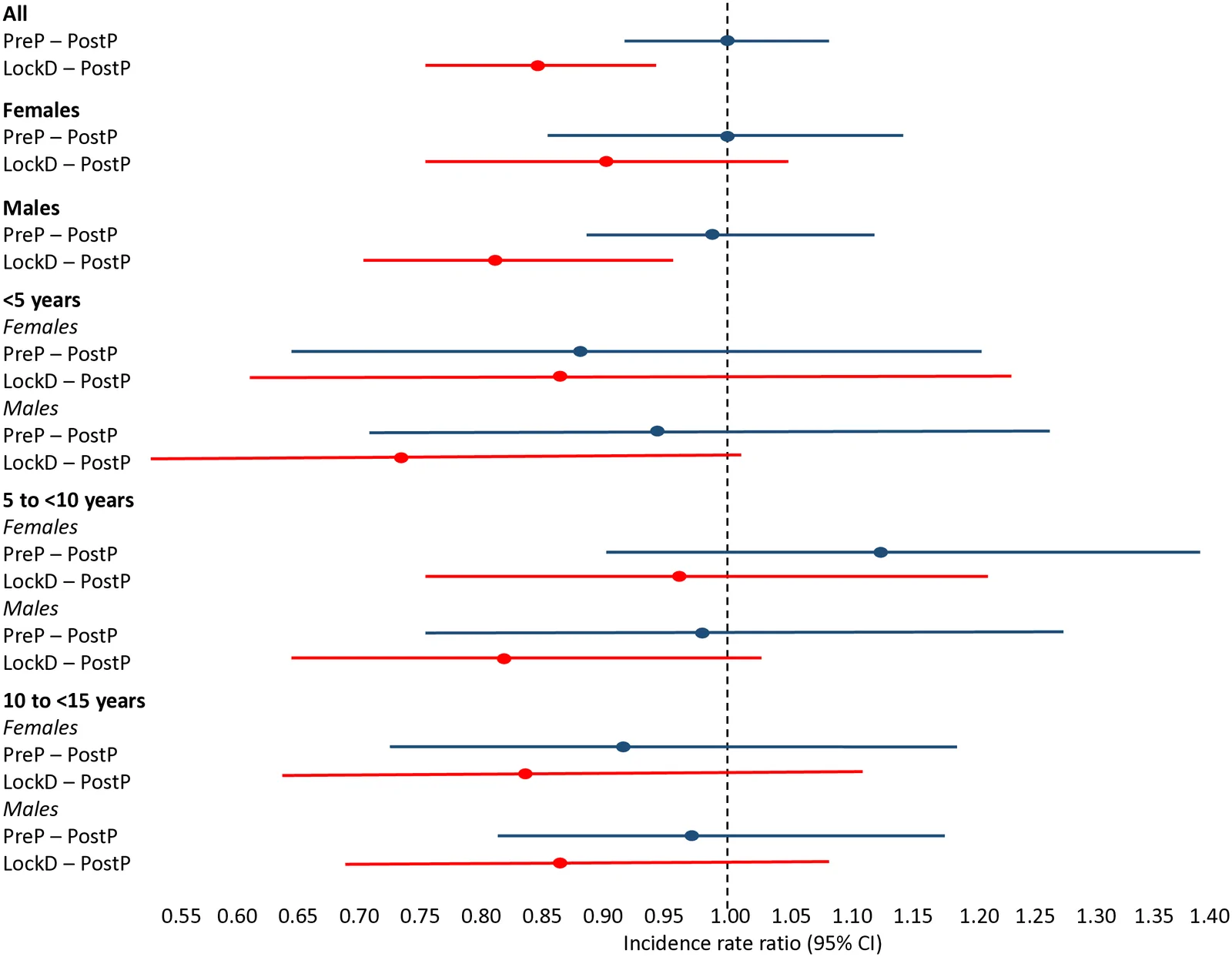
Incidence rate ratios of type 1 diabetes stratified by sex and age group across study periods. The forest plot displays incidence rate ratios (IRRs) with their corresponding 95% confidence intervals (CIs) for Finnish children aged <15 years. Blue markers and horizontal bars represent the comparison between the pre-pandemic reference period (PreP: three 18-month blocks, 2014–2019) and the post-lockdown period (PostP: September 2021–December 2022). Red markers and horizontal bars represent the comparison between the societal lockdown period (LockD: March 2020–August 2021) and the post-lockdown period (PostP). The vertical dashed line indicates an IRR of 1.00 (no difference in incidence rates). Estimates are provided for the entire cohort (“All”) and stratified by sex (Females, Males) across three specific age categories (<5 years, 5 to <10 years, and 10 to <15 years).

### Demographic, clinical, and metabolic characteristics and HLA genotypes

Children diagnosed with type 1 diabetes after the lockdown were older (8.57 years *versus* 8.17 years; *p* = 0.031), more likely to have severe ketoacidosis (9.4% *versus* 5.9%; *p* = 0.007), ketoacidosis (27.0% *versus* 22.6%; *p* = 0.053), and had had a shorter symptomatic period before the diagnosis compared to children diagnosed with type 1 diabetes before the lockdown (*p* = 0.011) (Tables 2 and S2). When comparing those diagnosed after the lockdown to the other two cohorts, we found no evidence of differences in other clinical characteristics, sex distribution, number of family members affected by type 1 diabetes, consciousness, blood pH, and plasma β-hydroxybutyrate concentration (Table 2). Plasma glucose and HbA_1c_ concentrations were neither significantly different in children diagnosed after the lockdown compared to the other two cohorts (Table 2). The proportion of children with HLA risk genotypes was found to be similar in the three study cohorts (S3 Table).

**Table 2:** Characteristics of children with type 1 diabetes diagnosed before (three 18-month terms 2014–2019), during (03/2020–08/2021) and after (09/2021–12/2022) the lockdown in Finland.

					*P* value, age- and/or sex-adjusted^a^	
***Demographic and clinical data***	**N respondents**	**Before lockdown (*n* = 2,096)**	**During lockdown (*n* = 785)**	**After lockdown (*n* = 586)**	**Before vs. After**	**During vs. After**
**Sex**	3,467				.712	.359
Males, *n* (%; 95% CI)		1,185 (56.5%; 54.4,58.6)	458 (58.4%; 54.9,64.7)	330 (56.3%; 52.2,60.4)		
Females, *n* (%; 95% CI)		911 (43.5%; 41.4,45.6)	327 (41.7%; 38.3,45.1)	256 (43.7%; 39.6,47.8)		
Age at diagnosis, years, estimated mean (95% CI)	3,426	8.17 (8.01,8.34)	8.37 (8.10,8.64)	8.57 (8.25,8.89)	.031	.342
Father with type 1 diabetes, *n* (%; 95% CI)	3,426	123 (5.9%; 4.9,7.0)	45 (5.7%; 4.3,7.6)	33 (6.1%; 4.2,8.4)	.797	.795
Mother with type 1 diabetes, *n* (%; 95% CI)	3,426	64 (3.1%; 2.4,3.9)	19 (2.4%; 1.6,3.7)	15 (2.8%; 1.6,4.5)	.809	.700
Sibling with type 1 diabetes, *n* (%; 95% CI)	3,426	115 (5.5%; 4.6,6.5)	36 (4.6%; 3.3,6.3)	21 (3.9%; 2.4,5.8)	.115	.508
Family members with type 1 diabetes, *n* (%; 95% CI)	3,426				.227	.856
0		1805 (86.1%; 84.6,87.5)	696 (88.7%; 86.3,90.7)	481 (88.3%; 85.3,90.8)		
1		271 (12.9%; 11.6,14.4)	76 (9.7%; 7.8,12.0)	60 (11.0%: 8.5,13.9)		
2		18 (0.9%; 0.5,1.4)	12 (1.5%; 0.9,2.7)	4 (0.7%; 0.2,1.9)		
3		2 (0.1%; 0.0,0.3)	0 (0%; 0.0,0.5)	0 (0%; 0.0, 0.1)		
4		0 (0.0%; 0.0,0.2)	1 (0.1%; 0.0,0.7)	0 (0.0%; 0.0,0.1)		
***Metabolic decompensation at diagnosis***						
**Duration of symptoms**	3,231				.011	.088
No symptoms, *n* (%; 95% CI)		65 (3.3%; 2.6,4.2)	37 (5.1%; 3.4,60.9)	38 (7.2%; 5.2,9.8)		
<1 week		344 (17.4%; 15.8,19.2)	123 (16.8%; 14.3,19.7)	89 (17.0%; 13.8,20.4)		
<4 weeks		1,173 (59.4%; 57.2,61.6)	425 (58.1%; 54.5,61.6)	308 (58.7%; 54.3,62.9)		
>4 weeks		392 (19.9%; 18.2,21.7)	147 (20.1%; 17.3,23.1)	90 (17.1%; 14.0,20.6)		
Plasma glucose, mmol/L, estimated mean (95% CI)	3,210	24.9 (24.5,25.4)	23.7 (23.0,24.4)	24.8 (23.9,25.7)	.801	.062
Ketoacidosis, *n* (%; 95% CI)	3,257	448 (22.6%; 20.8,24.5)	228 (30.8%; 27.6,34.2)	144 (27.0%; 23.3,30.9)	.053	.117
Severe ketoacidosis, *n* (%; 95% CI)	3,257	116 (5.9%; 4.9,7.0)	65 (8.8%; 6.9,11.0)	50 (9.4%; 7.0,12.2)	.007	.771
Impaired consciousness, *n* (%; 95% CI)	3,202	131 (6.6%; 5.6,7.7)	56 (7.5%; 5.8,9.6)	31 (6.7%; 4.6,9.4)	.930	.603
pH, median (IQR)	3,231	7.37 (7.31,7.41)	7.36 (7.26,7.40)	7.37 (7.28,7.41)	.194	.147
HbA_1c_, mmol/mol, median (IQR)	3,021	92.0 (77.0,114.0)	97.0 (78.0,119.0)	96.0 (77.0,199.0)	.249	.392
HbA_1c_, %, estimated mean (95% CI)	3,021	10.8 (10.7,10.9)	11.1 (10.9,11.3)	10.9 (10.7,11.1)	.527	.105
β-hydroxybutyrate, mmol/L, median (IQR)	3,122	2.00 (0.40,5.40)	2.60 (0.60,6.10)	2.30 (0.52,5.70)	.385	.394
***Autoantibodies***						
Glutamic acid decarboxylase antibodies, *n* (%; 95% CI)	2,677	1,036 (65.0%; 62.6,67.3)	426 (73.1; 69.3,76.6)	336 (67.3%; 63.0,71.4)	.553	.017
Glutamic acid decarboxylase antibodies, RU, median (IQR)	1,794	33.5 (13.4,98.5)	38.3 (12.9,114.3)	45.5 (16.8,129.2)	.004	.194
Islet antigen 2 antibodies, n (%; 95% CI)	2,677	1,166 (73.1%; 70.9,75.2)	434 (74.4%; 70.7,77.9)	397 (78.9%; 75.1,82.4)	.010	.079
Islet antigen 2 antibodies, RU, median (IQR)	1994	100.1 (42.6,130.7)	106.9 (53.5,131.9)	74.7 (13.7,127.7)	<.001	<.001
Insulin autoantibodies, *n* (%; 95% CI)	2,494	870 (56.6%; 54.1,59.1)	313 (57.4%; 53.2,61.6)	237 (57.5%; 52.6,62.4)	.309	.994
Insulin autoantibodies, RU, median (IQR)	1,418	6.1 (3.1,13.4)	6.1 (3.2,13.7)	4.7 (2.7,13.2)	.323	.244
Zinc transporter 8 antibodies, *n* (%; 95% CI)	2,677	1,104 (69.2%; 66.9,71.5)	421 (72.2%; 68.4,75.8)	349 (69.9%; 65.7,73.9)	.869	.265
Zinc transporter 8 antibodies, RU, median (IQR)	1874	11.0 (2.8,39.6)	15.0 (3.8,47.4)	12.6 (3.2,48.2)	.351	.269
Autoantibody negative, *n* (%; 95% CI)	2,675	61 (3.8%; 2.9,4.9)	19 (3.3%; 2.0,5.0)	21 (4.2%; 2.6,6.4)	.838	.414
Positivity for multiple autoantibodies, *n* (%; 95% CI) (≥2)	2,676	1,342 (84.1%; 82.3–85.9)	508 (87.1%; 84.1,89.8)	444 (89.2%; 86.1,91.8)	.013	.531
Number of detectable autoantibodies, median (IQR)	2,494	3 (2,3)	3 (2,4)	3 (2,4)	1.000	1.000

^a^Statistical tests used to determine *P* values: **Linear regression analysis:** Used for age at diagnosis and plasma glucose. **Quantile regression analysis:** Used to assess medians for HbA1c (mmol/mol and %), blood pH, plasma β-hydroxybutyrate, and all relative units (RU) of autoantibodies (Glutamic acid decarboxylase, Islet antigen 2, Insulin, Zinc transporter 8), as well as the number of detectable autoantibodies. **Logistic regression analysis:** Used for dichotomous response variables including sex, family history of type 1 diabetes (father, mother, sibling), ketoacidosis, severe ketoacidosis, impaired consciousness, autoantibody positivity (individual markers, multiple markers ≥2), and autoantibody negativity. **Multinomial logistic regression analysis:** Used for categorical variables with multiple categories, including duration of symptoms and the total number of family members with type 1 diabetes.

### Autoantibodies

Children diagnosed with type 1 diabetes after the lockdown tested positive for GADA less frequently than those diagnosed during the lockdown (67.3% *versus* 73.1%, *p =* 0*.*017; Table 2). The frequency of GADA positivity was similar after the lockdown compared to the time before lockdown. The study participants tested positive for IA-2A more often after the lockdown than before the lockdown (78.9% *versus* 73.1%, *p* = 0.010). The GADA levels were higher and IA-2A levels lower among those diagnosed with type 1 diabetes after the lockdown compared to children diagnosed before the lockdown (GADA: 45.5 RU *versus* 33.5 RU, *p* = 0.004; IA-2A: 74.7 RU *versus* 100.1 RU, *p* < 0.001). Furthermore, IA-2A levels were lower among those diagnosed after the lockdown compared to children diagnosed during the lockdown (74.7 RU *versus* 106.9 RU, *p <* 0.001). The frequency of positivity and the levels of ZnT8A did not differ between the study cohorts. Children diagnosed with type 1 diabetes after the lockdown period tested positive for multiple (≥2) autoantibodies more often compared to children that were diagnosed before the lockdown (89.2% *versus* 84.1%, *p* = 0.013). The frequency of autoantibody negativity and the number of detectable autoantibodies did not differ between the study cohorts.

### SARS-CoV-2 antibodies

SARS-CoV-2 antibodies were analyzed in 489 index cases diagnosed after the lockdown (83.8%). Of these, 317 (64.8%) tested positive for SARS-CoV-2 spike (S) antibodies by an enzyme-linked immunosorbent assay (ELISA) (S2 Fig), out of whom 303 also scored positive (titer ≥20) for neutralizing antibodies. The seroprevalence increased during the study period both due to infections and vaccinations, so that the vast majority (87.0%) of the children sampled in December 2022 had developed SARS-CoV-2 S antibodies. Among the tested index cases, 90 (18.4%) had IgG antibodies to SARS-CoV-2 nucleoprotein (*N*) (36.4%, *n* = 178, when borderline results typically seen after a few months from infection were also considered). The serological profile indicated a likely past infection in 201 (41.1%, strict criteria) or 252 (51.5%, loose criteria) children (S1 Table and S3 Fig). Altogether, 71% (*n* = 347) of the sampled children were unvaccinated or had received a vaccination <2 weeks before sample collection, comprising 32% (*n* = 153) of children with no sign of infection with any criteria; and 37% (181) or 34% (*n* = 167) with a serological profile indicating past infection with loose or strict criteria, respectively (S4 Fig). In addition, 2% (*n* = 9) or 5% (*n* = 23), respectively, had an ambiguous infection status. On the other hand, 29% of the children had been vaccinated before the diagnosis of type 1 diabetes, comprising 15% (*n* = 71) with serological evidence of a previous infection, while 14% (*n* = 70) had an ambiguous infection status based on loose criteria. These figures were 7% and 22% using the strict criteria, respectively. The ambiguous infection status generally resulted from not being able to distinguish vaccine- and infection-induced antibody responses in some vaccinated individuals. A major peak in N-antibody positivity occurred paralleling the Omicron variant surge in spring 2022 (S5 Fig). In December 2022, at the end of the study period an antibody profile attributable to a SARS-CoV-2 infection was observed in 77.4% (24/31, strict criteria) or 83.9% (26/31, loose criteria) of the children sampled in that period.

## Discussion

The lockdown represents a peak in type 1 diabetes incidence in Finland, not only compared to the time before lockdown, but also afterwards. Our study shows that the incidence of type 1 diabetes decreased by 15% among Finnish children during the first 16 months after the COVID-19 lockdown. The incidence of type 1 diabetes increased by an equal percentage during the initial 18-month lockdown period compared to the reference time before the COVID-19 pandemic.

After 2006 there has been a declining overall trend in the type 1 diabetes incidence in Finnish children until year 2020 and the COVID-19 lockdown. Considering this, a sudden 16% increase incidence during the lockdown followed by an equally sudden 15% decrease after the lockdown is striking. In fact, such an increase or decrease has never been detected during the whole history of FPDR data collection. Likewise, studies from Italy and Germany have reported that the increase in type 1 diabetes incidence rates observed in 2020 and 2021 did not persist into 2022 [2,3].

Our findings contradict the conception that the increase in the incidence of type 1 diabetes observed during the initial COVID-19 pandemic would be directly triggered by SARS-CoV-2 [5]. Firstly, we observed diametrically opposed changes in the incidence of type 1 diabetes and in the frequency of infection-induced SARS-CoV-2 antibodies in our research (Fig 1). Secondly, as SARS-CoV-2 was shown to be extremely rare in Finnish children during the lockdown, we can relatively reliably rule out COVID-19 infection as the causative agent of the observed increase in the incidence rate. Likewise, studies from the USA and Denmark showed no association between SARS-CoV-2 infections and type 1 diabetes [7,8]. In addition, no increased islet autoimmunity or type 1 diabetes was observed in a study of adolescents carrying genetic susceptibility to type 1 diabetes [19]. However, in young children with strong genetic risk for type 1 diabetes, SARS-CoV-2 antibodies have been associated with the development of islet autoantibodies in two European studies [9,10]. Furthermore, the rate of progression to clinical type 1 diabetes after COVID-19 infection was significantly accelerated in a sub-cohort of 509 children with positivity for multiple islet autoantibodies in a German study [11].

Indirect mechanisms may explain the repeatedly observed increase of type 1 diabetes incidence during the early pandemic [1,6]. For example, lockdowns have been associated with changes in physical activity [20], nutrition [21], weight [22], mental health [23], sleep [24] and time spent using electronic devices [25]. Furthermore, the lockdown resulted in substantially reduced human contacts. Some previous studies have observed a reduced risk of type 1 diabetes in environments with high social mixing [26,27]. In addition, the incidence of type 1 diabetes has been reported to be inversely associated with population density in a meta-analysis that combined 18 studies in 14 countries [28].

Our findings are in line with the hygiene hypothesis stating that increased exposure to childhood infections may provide some protection from type 1 diabetes in children [29]. In addition to social distancing, the pandemic response in Finland eventually included recommendations for face mask usage alongside public health campaigns for frequent hand washing and hand sanitizer use. All these actions have been shown to reduce the circulation of pathogens. National lockdowns and school closures in Finland immediately suppressed all respiratory pathogens except SARS-CoV-2 among the pediatric population [30]. The rate of most acute childhood infections decreased by 20%–90% during the first 12 months of the pandemic in an English study [31]. In Finland, a strong respiratory syncytial virus infection peak occurred soon after the lockdown ended [32]. The reopening of society also led to an increased frequency of COVID-19 infections in Finnish children in the autumn 2021 [33]. In line with the hygiene hypothesis, type 1 diabetes incidence decreased rapidly as viral infections became more common. While the opposing trends of type 1 diabetes incidence and SARS-CoV-2 antibody prevalence suggest an indirect pathway, the exact underlying mechanisms remain speculative. Factors such as the hygiene hypothesis or reduced lockdown exposure to other seasonal viruses cannot be directly proven by our data.

The frequency of autoantibody-negative type 1 diabetes was similar in our three study cohorts, which indicates that the higher incidence of type 1 diabetes during the lockdown is due to an immune-mediated mechanism. This result is in line with some population-based German studies [34,35]. Positivity for GADA was less frequent among those diagnosed after the lockdown period compared to the lockdown cohort in our study. In our previous study, the frequency of GADA positivity had increased during the lockdown compared to the time before lockdown [6]. Positivity for GADA has been associated with slower progression to clinical type 1 diabetes [36]. Children potentially progressed to clinical disease more swiftly during the lockdown than before lockdown, leading to a lower frequency of GADA positivity after the lockdown.

The decreased incidence rate of type 1 diabetes after the lockdown was observed for both sexes, although this finding was statistically significant for males only. The higher proportion of males among children with newly diagnosed type 1 diabetes provides more statistical power to analyze changes in disease rate among males. The frequency of severe DKA increased during the lockdown and remained high after the lockdown. This may reflect parents’ caution to seek medical care during the pandemic. The duration of symptoms was, however, significantly shorter and children were older after the lockdown compared to the time before lockdown. We have earlier reported that teens and children <2 years of age had the highest incidence of DKA at the time of diagnosis [12].

Like all research, our study has some strengths and limitations. First, our study covered practically the whole Finnish population of children with newly diagnosed type 1 diabetes. Secondly, we analyzed SARS-CoV-2 antibodies with several different methods in all available samples, and we verified the results of the analyses by comparing them to the vaccination register data. Nevertheless, distinguishing between antibodies from natural infection (anti-spike and anti-nucleocapsid) and vaccination (anti-spike) presents some challenges and resulted in ambiguous infection status in some cases. Study limitations include a relatively short follow-up period after the lockdown and the lack of explicit modeling of the underlying long-term secular trend in type 1 diabetes incidence. Another limitation is that the research was conducted only in Finland and to make any generalizations the study should be repeated in other countries. We adjusted our analyses for sex and age at diagnosis. Regardless, residual confounding due to some unknown or unmeasured variables can never be excluded in an observational study. FPDR is widely considered exceptionally complete and high in coverage. Nevertheless, there is no way to rule out the possibility that there were differences in the recording of type 1 diabetes cases during the different study periods.

Our study shows that a wave in the incidence of type 1 diabetes in children was observed after the onset of the COVID-19 pandemic. This included a peak in the incidence during the lockdown followed by a subsequent decline after the opening of the society. However, this reversion toward historical levels does not necessarily establish a return to the expected historical trajectory, nor can it fully distinguish a transient change from temporal compensation superimposed on the background secular trend. The contrasting patterns between type 1 diabetes incidence and SARS-CoV-2 antibody prevalence during and after the lockdown suggest an indirect rather than a direct role of the virus in triggering the observed changes in incidence rate. Together with the previous findings, our observations suggest that the SARS-CoV-2 virus is rather indirectly involved in the increase of type 1 diabetes rate than directly causing type 1 diabetes. The lockdown of the society accompanied with a strongly reduced rate of most acute infections may explain the increased incidence of type 1 diabetes. Our findings illustrate that environmental changes can rapidly modify the incidence of type 1 diabetes and suggest that exogenous factors play a crucial role in the manifestation of clinical disease.

## Supporting information

S1 Checklist STROBE Checklist. This checklist was obtained from the STROBE Statement website (https://www.strobe-statement.org/) and is used under the terms of the Creative Commons Attribution 4.0 International License (CC BY 4.0).
(DOCX)

S1 Protocol Study protocol. (DOCX)

S1 Text The Finnish Pediatric Diabetes Register. (DOCX)

S2 Text Lockdown measures in Finland. (DOCX)

S3 Text Analysis of islet autoantibodies. (DOCX)

S4 Text Analysis of SARS-COV-2 antibodies. (DOCX)

S1 Table Criteria used to define the SARS-CoV-2 infection status in children diagnosed with type 1 diabetes (DOCX)

S2 Table Characteristics of children with type 1 diabetes diagnosed before (three 18-month terms 2014–2019), during (03/2020–08/2021) and after (09/2021–12/2022) the lockdown in Finland, unadjusted analyses. (DOCX)

S3 Table HLA class II genotypes of children with type 1 diabetes diagnosed before (three 18-month terms 2014-2019), during (03/2020-08/2021) and after (09/2021-12/2022) the lockdown. Data shown as n (%, 95% CI), x* ≠ DR4-DQ8 or DR3-DQ. (DOCX)

S1 Fig Serum sample collection and SARS-CoV-2 antibody status in type 1 diabetes cases diagnosed during the lockdown (03/2020–08/2021) in Finland. (DOCX)

S2 Fig Serum sample collection and SARS-CoV-2 antibody status in type 1 diabetes cases diagnosed after the lockdown (09/2021–12/2022) in Finland. Only S-positive samples were further tested for neutralizing antibodies against the variants they may have been exposed to (considering the timing of the sampling and the time when each variant was circulating). Most samples that were positive in the neutralization test were positive against multiple variants.
(DOCX)

S3 Fig SARS-CoV-2 infection status of type 1 diabetes cases diagnosed during (03/2020–08/2021) and after the lockdown (09/2021–12/2022) in Finland. Definition of each criteria for the after-lockdown cohort is explained in S1 Table.
(DOCX)

S4 Fig SARS-CoV-2 infection and vaccination status in Finnish children diagnosed with type 1 diabetes after the lockdown (09/2021–12/2022). The status is classified as ‘infection’ (has had SARS-CoV-2 infection at some point in the past), ‘no infection’ (no evidence of SARS-CoV-2 infection in the past), or ‘ambiguous’ (could not be determined). Percentages (numbers of observations). Two different classification criteria (strict and loose, see S1 Table) were used, both leading to same overall conclusions. Children who had received vaccination less than 14 days before sampling (*n* = 4, unclear whether the vaccine had yet had time to induce an antibody response) are not included.
(DOCX)

S5 Fig Prevalence of SARS-CoV-2 antibodies and vaccination rates in Finnish children diagnosed with type 1 diabetes after the lockdown between September 1, 2021 and December 31, 2022. The median for the interval between the diagnosis and the collection of samples was 6 days (range 1–29 days). The decreasing trend of IgG N antibodies over time is similar with both cut offs despite the different prevalence values and agrees with the peak in cases in the overall Finnish population in early 2022. Since the vaccine was based on the S protein, antibodies against N protein result from a COVID-19 infection. The increase in prevalence of IgG S antibodies is due both to vaccination and to COVID-19 infections. As non-Wuhan stains were circulating during the study period and the vaccine used in the majority of the vaccinated children was based on the Wuhan strain, a higher titer (at least 4 times higher in this figure) against non-Wuhan strain is likely attributed to a previous COVID-19 infection.
(DOCX)

## Abbreviations

CI: confidence interval
DKA: Diabetic ketoacidosis
ELISA: enzyme-linked immunosorbent assay
FPDR: Finnish Pediatric Diabetes Register
GADA: Glutamic acid decarboxylase antibodies
HLA: human leukocyte antigen
IAA: Insulin autoantibodies
IA-2A: Islet antigen 2 antibodies
IRR: incidence rate ratios
LLM: Large Language Model
PY: person-years
RU: relative units
ZnT8A: Zinc transporter 8 autoantibodies
