# Supplementary material for: Trends in the incidence of childhood type 1 diabetes after the COVID-19 lockdown in Finland: An observational study
Source: PLoS Med. 2026 Nov 2;23(11):e1005288. doi: 10.1371/journal.pmed.1005288 (PMC13759143; doi:10.1371/journal.pmed.1005288)
Supplement: Supplementary file 2 [file pmed.1005288.s002.docx]

**S1 Study Protocol.**

Professor Mikael Knip

University of Helsinki

**An application to the Ethics Committee of the Helsinki and Uusimaa Hospital District**

**in relation to the research project: COVID-19 pandemic and risk of type 1 diabetes in Finnish children**

***I Background and state of the art***

Type 1 diabetes (T1D) is the most common serious chronic disease in children after allergies and asthma [1]. The incidence of T1D is increasing at an annual rate of about 3% [2]. It is expected that insulin will be difficult to access and afford in the future, especially in low-income countries [1]. Currently the lifetime costs of one subject with T1D diagnosed in childhood have been estimated to be about one million euros [1]. Furthermore, treatment of T1D requires ongoing glucose monitoring and causes continuous stress due to therapy, particularly for young children. Reducing T1D incidence would decrease personal stress and suffering of millions of children and their families as well as lead to major financial savings globally.

T1D is an autoimmune disease where the pancreatic insulin-producing beta cells are destroyed by the immune system. It is likely that genetically susceptible individuals will not develop T1D without an external trigger that initiates the autoimmune response [3]. Although the autoimmune pathophysiology of T1D has been extensively studied, the understanding of the triggering environmental factors remains elusive [3].

The hygiene hypothesis states that the increase in incidence of T1D has resulted from reduced microbial exposure due to increased hygiene [4,5]. Contacts to a diverse microbial community early in life are proposed to activate regulatory pathways in the immune system. This process prevents the development of T1D-associated autoimmunity. The number of social interactions is widely used as an estimate of microbial exposure of an individual [e.g., 6-8].

Coronavirus disease 2019 (COVID-19) and subsequent social distancing could be used as a proxy for infections to test the hygiene hypothesis. The pandemic has been associated with increased T1D incidence in an extensive meta-analysis that included 42 studies and 103 000 youths [9]. Knip et al. [10] found that only 0.9% of those diagnosed for T1D during the pandemic tested positive for infection-induced SARS-CoV-2 antibodies in Finland. Thereby, the observed increase in T1D incidence of 16% during the first 18 months of the pandemic in Finland cannot be attributed to a direct effect of SARS-CoV-2 but may rather be a consequence of the lockdown of society and social distancing.

***II Research goals***

The general goal of this research project is to assess whether social interactions are associated with the risk of T1D using the hygiene hypothesis as the theoretical framework and SARS-CoV-2 pandemic lock-down and daycare as proxy for the number of social interactions.

More specifically the objectives are:

To compare the incidence of T1D *after* the COVID-19 pandemic related daycare and school closures ended to the time when lock-down related school and daycare closures were implemented in Finland.

***III Hypotheses***

To achieve the goals of this project the following hypotheses based on the hygiene hypothesis will be tested:

*Hypothesis:* School and daycare closures are associated with increased incidence of T1D.

***IV Research implementation***

The incidence of T1D after the lockdown (09/2021-12/2022) is compared to the incidence during the lockdown (03/2020-08/2021) and before the lockdown (three 18-month terms 2014-2019) using the Finnish Pediatric Diabetes Register (FPDR) data The FPDR was initiated in 2002 and covers more than 90% of all children diagnosed with diabetes during and after that year. The parents have given their informed consent to the participation of their child in the FPDR. Demographic and clinical data are collected at the time of diagnosis. The participants donate biological samples [serum, EDTA blood and peripheral blood molecular cells (PBMCs)] taken close to the diabetes diagnosis to the sample repository linked to the FPDR

We will compare the clinical characteristics, signs of islet autoimmunity (GADA, IAA, IA-2A and ZnT8A autoantibodies), and frequency of risk-associated HLA genotypes in affected children during the three time periods listed above. Children under 15 years of age are included.

The following response variables will be analyzed:

Age at diagnosis (years)

Sex (female/male)

First degree relative with type 1 diabetes (father, mother, sibling, none)

Duration of symptoms (<1 week, <4 weeks, >4 weeks)

Plasma glucose (mmol/L)

Ketoacidosis, frequency

Severe ketoacidosis, frequency

Impaired consciousness, n/total

plasma pH

HbA_1c,_ mmol/mol

β–hydroxybutyrate, mmol/L

Autoantibody positivity and concentration in relative units (RU)

- GADA
- IA2A
- IAA
- ZnT8A

Autoantibody negativity, frequency

Positivity for multiple autoantibodies, frequency

Number of detectable autoantibodies (0, 1, 2, 3, 4)

HLA-risk genotype category (high risk, moderately increased risk, slightly increased risk, neutral, slightly decreased risk, strongly decreased risk)

HLA genotype

- DR3-DQ2 and DR4-DQ8DR3-DQ2, frequency
- DR3-DQ2 homozygote, frequency
- DR4-DQ8 homozygote, frequency
- DR3-DQ2 positive, frequency
- DR4-DQ8 positive, frequency

In addition ELISA and pseudovirus neutralization assays based on the latest advances in molecular medicine are used to assess whether SARS-CoV-2 triggers T1D in children (positivity for SARS-CoV-2 antibodies in the serum samples of T1D patients compared to healthy peers).

Diabetes-associated autoantibodies are analyzed in the PEDIA laboratory, University of Helsinki, the HLA genotypes are defined in the Immunogenetics Laboratory, Institute of Biomedicine, University of Turku. SARS-CoV-2 antibodies are analyzed in the Department of Virology, University of Helsinki. Data will be organized and analyzed for the specific purposes of each research paper. Data will be stored in a secure online location managed by the Pediatric Diabetes Research Group (PEDIA). The data is only accessible by username and password.

***VI References***

1. Mobasseri M, Shirmohammadi M, Amiri T, et al. Prevalence and incidence of type 1 diabetes in the world: a systematic review and meta-analysis. Health Promot Perspect 2020; 10: 98-115.
2. Aanstoot HJ, Anderson B, Daneman D, et al. The global burden of youth diabetes: perspectives and potential. Pediatr Diabetes 2007; 8: 1-44.
3. Knip M, Veijola R, Virtanen SM, et al. Environmental triggers and determinants of type 1 diabetes. Diabetes 2005; 54: 125-136.
4. Strachan DP. Hay fever, hygiene, and household size. BMJ 1989; 299: 1259-60.
5. Bach JF, Chatenoud L. The hygiene hypothesis: an explanation for the increased frequency of insulin-dependent diabetes. Cold Spring Harb Perspect Med 2012; 2;4:a007799.
6. Schober E, Rami B, Waldhoer T. Small area variation in childhood diabetes mellitus in Austria: links to population density, 1989 to 1999. J Clin Epidemiol 2003; 56(3): 269-273.
7. McKinney PA, Okasha M, Parslow RC, et al. Early social mixing and childhood T1D mellitus: a case- control study in Yorkshire, UK. Diabet Med 2000; 3: 236-242.
8. Parslow RC, McKinney PA, Law GR, Bodansky HJ. Population mixing and childhood diabetes. Int J Epidemiol 2001; 30(3): 533-538.
9. D'Souza D, Empringham J, Pechlivanoglou P, Uleryk EM, Cohen E, Shulman R. Incidence of Diabetes in Children and Adolescents During the COVID-19 Pandemic: A Systematic Review and Meta-Analysis. JAMA Netw Open. 2023;6(6):e2321281.
10. Knip M, Parviainen A, Turtinen M**,** et al. SARS-CoV-2 and type 1 diabetes in children in Finland: an observational study. Lancet Diabetes Endocrinol. 2023;11(4):251-260.

.
