# Supplementary material for: Trends in the incidence of childhood type 1 diabetes after the COVID-19 lockdown in Finland: An observational study
Source: PLoS Med. 2026 Nov 2;23(11):e1005288. doi: 10.1371/journal.pmed.1005288 (PMC13759143; doi:10.1371/journal.pmed.1005288)
Supplement: Supplementary file 4 [file pmed.1005288.s004.docx]

**S2 Text. Lockdown measures in Finland**

The lockdown period started on March 1, 2020 when social distancing regulations were put into use in Finland [1]. The distancing regulations were then adjusted several times depending on the prevailing availability in medical intensive care units, COVID-19 incidence statistics, new scientific observations about the effectiveness of different social distancing measures and the preferences of the local authorities and WHO guidelines [1] Some social distancing regulations were in place throughout the lockdown study period. August 31, 2021 was set as the ending point of the lockdown as social distancing measures were relieved in August-September 2021 in Finland. In addition, a new school year with classroom teaching started leading to more social contacts and increased infection rates among children in the autumn 2021 [2].

**References**

1. Karreinen S, Rautiainen P, Keskimäki I, Satokangas M, Viita-aho M, Tynkkynen LK. Pandemic preparedness and response regulations in Finland: Experiences and implications for post-COVID-19 reforms. Health Policy. 2023;132:104802.

2. Kuitunen I, Artama M, Haapanen M, Renko M. Respiratory virus circulation in children after relaxation of COVID-19 restrictions in fall 2021-A nationwide register study in Finland. J Med Virol. 2022;94(9):4528-4532.
