# Supplementary material for: Trends in the incidence of childhood type 1 diabetes after the COVID-19 lockdown in Finland: An observational study
Source: PLoS Med. 2026 Nov 2;23(11):e1005288. doi: 10.1371/journal.pmed.1005288 (PMC13759143; doi:10.1371/journal.pmed.1005288)
Supplement: Supplementary file 5 [file pmed.1005288.s005.docx]

**S3 Text. Analysis of islet autoantibodies**

We analyzed autoantibodies to insulin (IAA), truncated glutamic acid decarboxylase (GADA 96-585), islet antigen 2 (IA-2A) and zinc transporter 8 (ZnT8A) with specific radiobinding assays [1]. We used cut-off limits for positivity of 1.57 relative units (RU) for IAA, 5.36 RU for GADA, 0.78 RU for IA-2A, and 0.50 RU for ZnT8 based on 372 healthy Finnish children. Our laboratory has taken part in the Islet Autoantibody Standardization Program (IASP) workshops in 2016, 2018, 2020 and 2023. The disease sensitivity of the IAA assay has ranged from 53 to 66% and the disease specificity from 92 to 94%. The corresponding values for the GAD antibody assay varied from 70 to 78% and 94 to 100%. The sensitivity for the IA-2A assay varied from 62 to 72% and that for the ZnT8A from 62 to 74%. The disease specificity for both assays remained at 100%. Only the antibody titers in positive samples were compared.

**References**

1. Knip M, Virtanen SM, Seppä K, Ilonen J, [Savilahti](https://pubmed.ncbi.nlm.nih.gov/?sort=date&term=Savilahti+E&cauthor_id=21067382) E, [Vaarala](https://pubmed.ncbi.nlm.nih.gov/?sort=date&term=Vaarala+O&cauthor_id=21067382) O, et al. Dietary intervention in infancy and later signs of beta-cell autoimmunity. N Engl J Med. 2010;363:1900–1908.
