# Supplementary material for: Trends in the incidence of childhood type 1 diabetes after the COVID-19 lockdown in Finland: An observational study
Source: PLoS Med. 2026 Nov 2;23(11):e1005288. doi: 10.1371/journal.pmed.1005288 (PMC13759143; doi:10.1371/journal.pmed.1005288)
Supplement: Supplementary file 6 [file pmed.1005288.s006.docx]

**S4 Text. Analysis of SARS-COV-2 antibodies**

The production of recombinant Spike (S) ELISA testing were done as described by Rusanen et al. [1] and based on the original S construct and protocol described by Amanat et al. [2] and Stadlbauer et al. [3]. In addition, the Abbott Alinity test for measuring IgG antibodies to Nucleoprotein (N not induced in the spike protein mRNA-based vaccines used in Finland) was performed according to the manufacturer's instructions at Helsinki University Hospital Diagnostic Centre with cut-offs defined as detailed below. For S-antibodies we set up an automated high-throughput ELISA on a 384-well plate format as follows: Plates (Nunc 384-well MaxiSorp, non-sterile, #460372 for S and R) were coated with purified antigens in 200 μl /well 0.05 M carbonate buffer (pH 9.6, Medicago, Sweden) overnight at 4°C, S protein 1 μg/ml, and antigen 1.5 μg/ml. For each 384-well plate with serum samples, six plates were coated with each antigen to give two replicate plates. After antigen coating, the wells were emptied with BioTek EL406 96 pin washer manifold, and 30 μl blocking buffer (3% non-fat milk in PBS with 0.1% Tween 20) was added to all wells with EL406 syringe pump. Blocking solution was incubated for 1h at room temperature, and the wells were washed once with 60 μl of PBST (PBS with 0.05% Tween 20) using EL406. Washing solution (PBST) was dispensed with a syringe pump. For serum sample dilution, 15μl of assay buffer (1% non-fat milk in PBS with 0.05% Tween 20) was added with CertusFlex. Serum samples were pre-diluted in concentration of 1/25 to assay buffer on 384 intermediate plate (Axygen 384-well Clear V-Bottom 240 μL Polypropylene DeepWell Not Treated Plate, #P -384-240SQ-C) pipetting with Biomek FXp 96 head (BeckmanCoulter). After washing three times with EL406 washer manifold with 80 µl of PBST, horseradish peroxidase (HRP) conjugated goat anti-human IgG-HRP 1/6000 was applied. After incubation for 1 h at room temperature plates were washed three times with 80 μl PBST as previously and then wells filled with Pierce™ ECL Western Blotting Substrate (#32106) using CertusFlex. After 10 min of incubation, the plates were read for luminescence BMG Pherastar FS, with measurement interval of 1 sec, and focal height 12.5 mm for Nunc plate. The assays have been evaluated to have a sensitivity of 100% in detecting IgG seroconversion more than 13 days after onset of illness in SARS-COV-2 RT-PCR positive COVID-19 patients [1].

The samples testing positive in the ELISA test were analysed for neutralizing capacity against the Wuhan, Delta and Omicron BA.1, BA.2, and BA.5 variants using lentiviral pseudotypes constructed as described [4,5]. Because of similar neutralization titers between wild-type and Alpha (B.1.1.7) variants [4], and the scarcity of the Alpha variant in the autumn 2021 and in children infected before this [6], Alpha variant was not used, whereas Delta and Omicron (BA.1, BA.2 and BA.5) variants were prevalent during the study period [5]. The sera were serially diluted (2-fold from 1:20 to 1:1280) with a final volume of 30 µL. An equal volume of the appropriately diluted pseudovirus stock was added to each well and incubated for 1 h at +37 ⁰C for neutralization to occur. While incubating, 293T ACE-2 cells, grown on a confluent monolayer in a T75 flask were detached using Trypsin-EDTA (0.25%, Gibco) and resuspended at 1 million cells/ml in media with 10% Fetal Bovine Serum (FBS), 100 IU penicillin, 100 ug/mL streptomycin, and 2 mM L-glutamine. After the neutralization incubation, 40 µL of cell suspension was added to each of the neutralization wells and the mixture was seeded on a black 96-well plate followed by incubation at +37 ⁰C in a 5% CO_2_ incubator. After 48 h, the media was replaced with 50μl of Renilla-Glo Luciferase assay reagent (Promega), reconstituted according to manufacturers’ specifications, and incubated for 10 min before measuring the luminescence on HIDEX sense microplate reader Version 1·12. The neutralizing (end-point) titers were computed as the serum dilution that resulted in an 80% reduction in luminescence as compared to the virus control (without serum).

**Calculation of cut off values for N-IgG test**

As Abbott Alinity N-IgG test values have been reported to wane and become negative gradually some months after the acute phase with the 1.4 cut off value recommended by the manufacturer [7,8]. An additional cut off vas determined by testing a panel of 100 serum samples collected before the pandemic. A cut-off of 0.43 was defined by adding two standard deviations to the mean value of the negative panel. Any result that was between 0.43 and 1.4 was considered borderline. With the 0.43 cut off, 2/100 samples gave a positive result, resulting in a false positivity rate of 2%. Cut off of 0.43 is also supported by earlier studies in pregnant women in Finland [9]. The half-life of (SARS-CoV-2 virus nucleoprotein) N antibodies in adults has been reported to be 116 days using this test [10].

**Determining the SARS-CoV-2 infection status**

To estimate the prevalence of children who had an antibody profile compatible with previous infection, we considered vaccination status, S- and N-IgG results, and antibody titers to different variants compared to the original Wuhan strain used in the vaccine (but no longer circulating during the study period) in in children diagnosed with type 1 diabetes after the lockdown in Finland. Pediatric vaccines were based on RNA of the Wuhan strain from August 2021 to September 2022, when “variant” RNA vaccines with Wuhan/BA.1 in a 50:50 ratio were introduced.

Due to the difficulties separating vaccine-induced and infection-induced antibody responses, two different criteria (strict and loose) were used to check whether they lead to the same overall conclusions. The following aspects were considered:

- Vaccination status of the patient at the time of sampling. Patient was considered vaccinated if they had received vaccine at least 14 days before sampling. Vaccination status of those who received the vaccine 0-14 days before sampling (4 children) was considered ambiguous.
- Results of S and N IgG tests.
- Comparison of neutralizing antibody titers between Wuhan stain (used in the vaccine) and other tested variants

**References**

1. Rusanen J, Kareinen L, Levanov L, Mero S, Pakkanen SH, Kantele A, et al. A 10-minute “mix and read” antibody assay for SARS-CoV-2. Viruses. 2021 Jan 20;13(2):143.
2. Amanat F, Stadlbauer D, Strohmeier S, Nguyen TH, Chromikova V, McMahon M, et al. A serological assay to detect SARS-CoV-2 seroconversion in humans. Nat Med. 2020;26:1033–1036.
3. Stadlbauer D, Amanat F, Chromikova V, Jiang K, Strohmeier S, Arunkumar GA, et al. SARS-CoV-2 seroconversion in humans: A detailed protocol for a serological assay, antigen production, and test setup. Curr Protoc Microbiol. 2020;57:e100.
4. Mäkelä AR, Uğurlu H, Hannula L, Kant R, Salminen P, Fagerlund R, et al. Intranasal trimeric sherpabody inhibits SARS-CoV-2 including recent immunoevasive Omicron subvariants. Nat Commun. 2023;14:1637.
5. Khawaja T, Kajova M, Levonen I, Pietilä JP, Välimaa H, Paajanen J, et al. Double-blinded, randomised, placebo-controlled trial of convalescent plasma for COVID-19: analyses by neutralising antibodies homologous to recipients' variants. Infect Dis (Lond). 2024;56:423–433.
6. Knip M, Parviainen A, Turtinen M, [But](https://pubmed.ncbi.nlm.nih.gov/?sort=date&term=But+A&cauthor_id=36958868) A, [Härkönen](https://pubmed.ncbi.nlm.nih.gov/?sort=date&term=H%C3%A4rk%C3%B6nen+T&cauthor_id=36958868) T, [Hepojoki](https://pubmed.ncbi.nlm.nih.gov/?sort=date&term=Hepojoki+J&cauthor_id=36958868) J, et al. SARS-CoV-2 and type 1 diabetes in children in Finland: an observational study. Lancet Diabetes Endocrinol. 2023;11:251-260.
7. Virtanen J, Uusitalo R, Korhonen EM, Aaltonen K, Smura T, Kuivanen S, et al. Kinetics of neutralizing antibodies of SARS-COV-2 patients tested using clinical D614G, B.1.1.7, and B.1.351 isolates in microneutralization assays. Viruses. 2021;13:996.
8. García-Carreras B, Hitchings MD, Johansson MA, Biggerstaff M, Slayton RB, Healy JM, et al. Accounting for assay performance when estimating the temporal dynamics in SARS-CoV-2 seroprevalence in the U.S. Nat Commun. 2023;14:2235.
9. Virtanen J, Korhonen EM, Salonen S, Vapalahti O, Sironen T, Jääskeläinen AJ. SARS-CoV-2 infections among pregnant women, 2020, Finland—Cross-testing of neutralization assays. J Med Virol. 2024;96:e29415.
10. Van Elslande J, Oyaert M, Lorent N, Vande Weygaerde Y, Van Pottelbergh G, Godderis L, et al. Lower persistence of anti-nucleocapsid compared to anti-spike antibodies up to one year after SARS-CoV-2 infection. Diagn Microbiol Infect Dis. 2022;103:115659.
