# Supplementary material for: Trends in the incidence of childhood type 1 diabetes after the COVID-19 lockdown in Finland: An observational study
Source: PLoS Med. 2026 Nov 2;23(11):e1005288. doi: 10.1371/journal.pmed.1005288 (PMC13759143; doi:10.1371/journal.pmed.1005288)
Supplement: Supplementary file 7 [file pmed.1005288.s007.docx]

**S1 Table.**  Criteria used to define the SARS-CoV-2 infection status in children diagnosed with type 1 diabetes after the lockdown (09/2021–12/2022) in Finland.

|  | Strict criteria | Loose criteria |
| --- | --- | --- |
| Has had SARS-CoV-2 infection at some point in the past | Positive with S and N IgG tests *or* | Positive or borderline with S and N IgG tests *or* |
|  | Neutralization titer against Delta or any Omicron variant at least 4 times the titer against the Wuhan strain *or* | Neutralization titer against Delta or any Omicron variant at least 2 times the titer against the Wuhan strain *or* |
|  | Not vaccinated and positive for S IgG and positive (titer => 20) in pseudoneutralization | Not vaccinated and positive/borderline for S IgG |
| No evidence of SARS-CoV-2 infection in the past | Negative with both S and N IgG tests | Negative with both S and N IgG tests |

SARS-CoV-2 infection status of children who did not fill any of the criteria was classified as ‘undefined’
