# Supplementary material for: Trends in the incidence of childhood type 1 diabetes after the COVID-19 lockdown in Finland: An observational study
Source: PLoS Med. 2026 Nov 2;23(11):e1005288. doi: 10.1371/journal.pmed.1005288 (PMC13759143; doi:10.1371/journal.pmed.1005288)
Supplement: Supplementary file 8 [file pmed.1005288.s008.docx]

**S2 Table.**  Characteristics of children with type 1 diabetes diagnosed before (three 18-month terms 2014–2019), during (03/2020–08/2021) and after (09/2021–12/2022) the lockdown in Finland, unadjusted analyses.

|  | | | | | ***P* value, unadjusted^a^** | |
| --- | --- | --- | --- | --- | --- | --- |
| ***Demographic and clinical data*** | **N respondents** | **Before lockdown (n=2096)** | **During lockdown (n=785)** | **After lockdown (n=586)** | **Before vs After** | **During vs After** |
| **Sex** | 3467 |  |  |  | .923 | .453 |
| Males, n (%; 95% CI) |  | 1185 (56.5%; 54.4–58.6) | 458 (58.4%; 54.9–64.7) | 330 (56.3%; 52.2–60.4) |  |  |
| Females, n (%; 95% CI) |  | 911 (43.5%; 41.4–45.6) | 327 (41.7%; 38.3–45.1) | 256 (43.7%; 39.6–47.8) |  |  |
| Age at diagnosis, years, mean (SD) | 3426 | 8.20 (3.86) | 8.41 (3.75) | 8.60 (3.79) | .033 | .370 |
| Father with type 1 diabetes, n (%; 95% CI) | 3467 | 123 (5.9%; 4.9–7.0) | 45 (5.7%; 4.3–7.6) | 33 (5.6%; 3.9–7.8) | .830 | .938 |
| Mother with type 1 diabetes, n (%; 95% CI) | 3467 | 64 (3.1%; 2.4–3.9) | 19 (2.4%; 1.6–3.7) | 15 (2.6%; 1.4–4.2) | .533 | .870 |
| Sibling with type 1 diabetes, n (%; 95% CI) | 3467 | 115 (5.5%; 4.6–6.5) | 36 (4.6%; 3.3–6.3) | 21 (3.6%; 2.2–5.4) | .066 | .359 |
| Family members with type 1 diabetes, n (%; 95% CI) | 3467 |  |  |  | .062 | .761 |
| 0 |  | 1805 (86.1%; 84.6–87.5) | 696 (88.7%; 86.3–90.7) | 522 (89.0%; 86.3–91.5) |  |  |
| 1 |  | 271 (12.9%; 11.6–14.4) | 76 (9.7%; 7.8–12.0) | 60 (10.2%: 7.9–13.0) |  |  |
| 2 |  | 18 (0.9%; 0.5–1.4) | 12 (1.5%; 0.9–2.7) | 4 (0.7%; 0.2–1·7) |  |  |
| 3 |  | 2 (0.1%; 0.0–0.3) | 0 (0.0%; 0.0–0.5) | 0 (0.0%; 0.0–0.1) |  |  |
| 4 |  | 0 (0.0%; 0.0–0.2) | 1 (0.1%; 0.0–0.7) | 0 (0.0%; 0.0–0.1) |  |  |
| ***Metabolic decompensation at diagnosis*** | | | | | | |
| **Duration of symptoms** | 3231 |  |  |  | .024 | .116 |
| No symptoms, n (%; 95% CI) |  | 65 (3.3%; 2.6–4.2) | 37 (5.1%; 3.4–6.9) | 38 (7.2%; 5.2–9.8) |  |  |
| <1 week |  | 344 (17.4%; 15.8–19.2) | 123 (16.8%; 14.3–19.7) | 89 (17.0%; 13.8–20.4) |  |  |
| <4 weeks |  | 1173 (59.4%; 57.2–61.6) | 425 (58.1%; 54.5–61.6) | 308 (58.7%; 54.3–62.9) |  |  |
| >4 weeks |  | 392 (19.9%; 18.2–21.7) | 147 (20.1%; 17.3–23.1) | 90 (17.1%; 14.0–20.6) |  |  |
| Plasma glucose, mmol/L, mean (SD) | 3229 | 24.9 (10.4) | 23.8 (8.8) | 24.7 (10.8) | .632 | .099 |
| Ketoacidosis, n (%; 95% CI) | 3257 | 448/1982 (22.6%; 20.8–24.5) | 228/741 (30.8%; 27.6–34.2) | 144/534 (27.0%; 23.3–30.9) | .035 | .141 |
| Severe ketoacidosis, n (%; 95% CI) | 3257 | 116/1982 (5.9%; 4.9–7.0) | 65/741 (8.8%; 6.9–11.0) | 50/534 (9.4%; 7.0–12.2) | .004 | .716 |
| Impaired consciousness, n (%; 95% CI) | 3223 | 131/1997 (6.6%; 5.6–7.7) | 56/744 (7.5%; 5.7–9.7) | 31/482 (6.4%; 4.4–9.0) | .919 | .466 |
| pH, median (IQR) | 3257 | 7.37 (7.31–7.41) | 7.36 (7.26–7.40) | 7.37 (7.29–7.41) | 1.000 | .064 |
| HbA_1c_, mmol/mol, median (IQR) | 3045 | 92·0 (77.0–114.0) | 97.0 (78.0–119.0) | 96.0 (77.0–199.0) | .035 | .684 |
| HbA_1c_, %, mean (SD) | 3045 | 10.8 (2.5) | 11.1 (2.6) | 10.9 (2.7) | .174 | .295 |
| β–hydroxybutyrate, mmol/L, median (IQR) | 3147 | 2.00 (0.40–5.40) | 2.60 (0.60–6.10) | 2.30 (0.51–5.70) | .310 | .387 |
| ***Autoantibodies*** | | | | | | |
| Glutamic acid decarboxylase antibodies, n (%; 95% CI) | 2677 | 1036 (65.0%; 62.6–67.3) | 426 (73.1; 69.3–76.6) | 332 (66.5%; 62.2–70.7) | .518 | .020 |
| Glutamic acid decarboxylase antibodies, RU, median (IQR) | 1794 | 33.5 (13.4–98.5) | 38.3 (12.9–114.3) | 45.5 (16.8–129.2) | .034 | .273 |
| Islet antigen 2 antibodies, n (%; 95% CI) | 2677 | 1166 (73.1%; 70.9–75.3) | 434 (74.4%; 70.7–77.9) | 394 (79.0%; 75.1–82.5) | .009 | .081 |
| Islet antigen 2 antibodies, RU, median (IQR) | 1997 | 100.1 (42.6–130.7) | 106.9 (53.5–131.9) | 74.6 (13.7–127.3) | <.001 | <.001 |
| Insulin autoantibodies, n (%; 95% CI) | 2495 | 870 (56.6%; 54.1–59.1) | 313 (57.4%; 53.2–61.6) | 235 (56.9%; 52.0–61.7) | .914 | .870 |
| Insulin autoantibodies, RU, median (IQR) | 1418 | 6.1 (3.1–13.4) | 6.1 (3.2–13.7) | 4.7 (2.7–13.2) | .008 | .055 |
| Zinc transporter 8 antibodies, n (%; 95% CI) | 2677 | 1104 (69.2%; 66.9–71.5) | 421 (72.2%; 6.4–75.8) | 345 (69.1%; 64.9–73.2) | .943 | .306 |
| Zinc transporter 8 antibodies, RU, median (IQR) | 1874 | 10.9 (2.8–39.6) | 15.0 (3.8–47.4) | 12.6 (3.2–48.2) | .465 | .424 |
| Autoantibody negative, n (%; 95% CI) | 2676 | 61 (3.8%; 2.9–4.9) | 19 (3.3%; 2.0–5.0) | 22 (4.4%; 2.8–6.6) | .554 | .322 |
| Positivity for multiple autoantibodies, n (%; 95% CI) (≥2) | 2677 | 1342 (84.1%; 82.3-85.9) | 508 (87.1%; 84.1–89.8) | 440 (88.2%; 85.0–90.9) | .024 | .571 |
| Number of detectable autoantibodies, median (IQR) | 2495 | 3 (2–3) | 3 (2–4) | 3 (2–4) | 1.000 | 1.000 |

${}^{a}$Statistical tests used to determine *P* values:

- **Linear regression analysis:** Used for age at diagnosis and plasma glucose.
- **Quantile regression analysis:** Used to assess medians for HbA1c (mmol/mol and %), blood pH, plasma $\beta$-hydroxybutyrate, and all relative units (RU) of autoantibodies (Glutamic acid decarboxylase, Islet antigen 2, Insulin, Zinc transporter 8), as well as the number of detectable autoantibodies.
- **Logistic regression analysis:** Used for dichotomous response variables including sex, family history of type 1 diabetes (father, mother, sibling), ketoacidosis, severe ketoacidosis, impaired consciousness, autoantibody positivity (individual markers, multiple markers $\geq$2), and autoantibody negativity.
- **Multinomial logistic regression analysis:** Used for categorical variables with multiple categories, including duration of symptoms and the total number of family members with type 1 diabetes.
