# Supplementary material for: Trends in the incidence of childhood type 1 diabetes after the COVID-19 lockdown in Finland: An observational study
Source: PLoS Med. 2026 Nov 2;23(11):e1005288. doi: 10.1371/journal.pmed.1005288 (PMC13759143; doi:10.1371/journal.pmed.1005288)
Supplement: Supplementary file 9 [file pmed.1005288.s009.docx]

**S3 Table.** HLA class II genotypes of children with type 1 diabetes diagnosed before (three 18-month terms 2014–2019), during (03/2020–08/2021) and after (09/2021–12/2022) the lockdown in Finland. Data shown as n (%, 95% Cl), x ⃰ ≠ DR4-DQ8 or DR3-DQ

|  |  |  |  |  | ***P* value, unadjusted** | | ***P* value, age and sex adjusted^a^** | |
| --- | --- | --- | --- | --- | --- | --- | --- | --- |
| **HLA II Genotype** | **N respondents** | Before lockdown (n=2096) | During lockdown (n=785) | After lockdown (n=586) | **Before vs After** | **During vs After** | **Before vs After** | **During vs After** |
| DR3-DQ2 and DR4-DQ8 | 2875 | 347 (20.2%; 18.3–22.1) | 128 (19.5%; 16.6–22.7) | 110 (22.2%; 18.6–26.1) | .326 | .263 | .262 | .248 |
| DR3-DQ2 and x ⃰ | 2874 | 234 (13.6%; 12.1–15.3) | 94 (13.5%; 11.1–16.4) | 62 (14.1%; 11.2–17.5) | .540 | .382 | .552 | .398 |
| DR4-DQ8 and x ⃰ | 2874 | 701 (40.7%; 38.4–43.0) | 255 (38.8%; 35.2–42.6) | 212 (42.8%; 38.4–47.3) | .398 | .170 | .412 | .174 |
| x ⃰⃰ | 2874 | 230 (13.4%; 11.8–15.0) | 109 (16.6%; 13.5–19.1) | 70 (14.1%; 11.2–17.5) | .653 | .256 | .714 | .246 |
| DR3-DQ2 homozygote | 2875 | 40 (2.3%; 1.7–3.1) | 16 (2.4%; 1.5–3.9) | 12 (2.4%; 1.3–4.2) | .900 | .986 | .897 | .998 |
| DR4-DQ8 homozygote | 2875 | 151 (8.8%; 7.5–10.2) | 55 (8.4%; 6.5–10.7) | 34 (6.9%; 4.8–9.5) | .176 | .341 | .158 | .325 |
| DR3-DQ2 positive | 2874 | 637 (37.0%; 34.7–39.3) | 238 (36.2%; 32.6–40.0) | 184 (37.0%; 32.9–41.6) | .942 | .742 | .843 | .703 |
| DR4-DQ8 positive | 2874 | 1202 (69.8%; 67.6–71.9) | 438 (66.7%; 63.0–70.2) | 351 (70.9% 66.7–74.9) | .636 | .125 | .601 | .126 |
| Risk group | 2874 |  |  |  | .169 | .164 | .173 | .155 |
| Highly increased | 585 | 347 (20.2%; 18.3–22.1) | 128 (19.5%; 16.6–22.7) | 110 (22.2%; 18.6–26.2) |  |  |  |  |
| Moderately  increased | 1067 | 640 (37.2%; 34.9–39.5) | 247 (37.6%; 34.0–41.3) | 180 (36.4%; 32.1–40.8) |  |  |  |  |
| Mildly increased | 678 | 420 (24.4%; 22.4–26.5) | 142 (21.6%; 18.6–24.9) | 116 (23.4%; 19.8–27.4) |  |  |  |  |
| Neutral | 442 | 260 (15.1%; 13.5–16.9) | 117 (17.8%; 15.1–20.9) | 65 (13.1%; 10.3–16.4) |  |  |  |  |
| Mildly protective | 75 | 44 (2.6%; 1.9–3.4) | 17 (2.6%; 1.6–4.1) | 14 (3.0%; 2.0–5.0) |  |  |  |  |
| Highly protective | 27 | 11 (0.6%; 0.4–1.1) | 6 (0.9%; 0.0–4.2)) | 10 (2.0%; 1.0–4.0) |  |  |  |  |

**^a^Statistical methods used to determine *P* values:**

- **Logistic regression analysis:** Used for individual HLA II Genotypes, homozygosity, and positivity rows, which represent dichotomous response variables (presence or absence of the specific genetic marker).
- **Multinomial logistic regression analysis:** Used for the *Risk group* variable, which contains multiple categorical classifications (Highly increased, Moderately increased, Mildly increased, Neutral, Mildly protective, Highly protective).
