# Supplementary material for: Trends in the incidence of childhood type 1 diabetes after the COVID-19 lockdown in Finland: An observational study
Source: PLoS Med. 2026 Nov 2;23(11):e1005288. doi: 10.1371/journal.pmed.1005288 (PMC13759143; doi:10.1371/journal.pmed.1005288)
Supplement: Supplementary file 10 [file pmed.1005288.s010.docx]

**S1 Figure.** Serum sample collection and SARS-CoV-2 antibody status in type 1 diabetes cases diagnosed during the lockdown (03/2020–08/2021) in Finland.
