# Supplementary material for: Trends in the incidence of childhood type 1 diabetes after the COVID-19 lockdown in Finland: An observational study
Source: PLoS Med. 2026 Nov 2;23(11):e1005288. doi: 10.1371/journal.pmed.1005288 (PMC13759143; doi:10.1371/journal.pmed.1005288)
Supplement: Supplementary file 11 [file pmed.1005288.s011.docx]

**S2 Fig.** Serum sample collection and SARS-CoV-2 antibody status in type 1 diabetes cases diagnosed after the lockdown (09/2021–12/2022) in Finland. Only S positive samples were further tested for neutralising antibodies against the variants they may have exposed to (considering the timing of the sampling and the time when each variant was circulating). Most samples that were positive in the neutralisation test were positive against multiple variants.
