# Supplementary material for: Trends in the incidence of childhood type 1 diabetes after the COVID-19 lockdown in Finland: An observational study
Source: PLoS Med. 2026 Nov 2;23(11):e1005288. doi: 10.1371/journal.pmed.1005288 (PMC13759143; doi:10.1371/journal.pmed.1005288)
Supplement: Supplementary file 12 [file pmed.1005288.s012.docx]

**S3 Fig.** SARS-CoV-2 infection status of type 1 diabetes cases diagnosed during (03/2020–08/2021) and after (09/2021–12/2022) the lockdown in Finland. Definition of each criterion for the after lockdown cohort is explained in supplementary table 1.
