# Supplementary material for: Trends in the incidence of childhood type 1 diabetes after the COVID-19 lockdown in Finland: An observational study
Source: PLoS Med. 2026 Nov 2;23(11):e1005288. doi: 10.1371/journal.pmed.1005288 (PMC13759143; doi:10.1371/journal.pmed.1005288)
Supplement: Supplementary file 13 [file pmed.1005288.s013.docx]

**S4 Fig.**

SARS-CoV-2 infection and vaccination status in Finnish children diagnosed with type 1 diabetes after the lockdown (09/2021–12/2022). The status is classified as ‘infection’ (has had SARS-CoV-2 infection at some point in the past), ‘no infection’ (no evidence of SARS-CoV-2 infection in the past), or ‘ambiguous’ (could not be determined). Percentages (numbers of observations). Two different classification criteria (strict and loose, see S1 Table) were used, both leading to same overall conclusions. Children who had received vaccination less than 14 days before sampling (n = 4, unclear vaccine had yet had time to cause an antibody response) are not included.
