# Supplementary material for: Trends in the incidence of childhood type 1 diabetes after the COVID-19 lockdown in Finland: An observational study
Source: PLoS Med. 2026 Nov 2;23(11):e1005288. doi: 10.1371/journal.pmed.1005288 (PMC13759143; doi:10.1371/journal.pmed.1005288)
Supplement: Supplementary file 14 [file pmed.1005288.s014.docx]

**S5 Fig.**

Prevalence of SARS-CoV-2 antibodies and vaccination rates in Finnish children diagnosed with type 1 diabetes after the lockdown between September 1, 2021 and December 31, 2022. The median for the interval between the diagnosis and the collection of samples was 6 days (range 1-29 days). The decreasing trend of IgG N antibodies over time is similar with both cut-offs despite the different prevalence values and is in concordance with the peak in cases in overall Finnish population with the Omicron wave in early 2022. Since the vaccine was based on S protein, antibodies against N protein result from a COVID-19 infection - however, they tend to wane with the method used (half-life 116 days (See Methods S4 Text). The increase in prevalence of IgG S antibodies is due both to vaccination and to COVID-19 infections. As non-Wuhan stains were circulating during the study period and the vaccine used in majority of the vaccinated children was based on Wuhan strain, a higher titer (at least 4 times higher in this figure) against non-Wuhan strain is likely attributed to a previous COVID-19 infection. The prevalence depicted for each month represents a sliding average of samples collected in that specific month, as well as the preceding and following month.
