# Supplementary material for: Trends in the incidence of childhood type 1 diabetes after the COVID-19 lockdown in Finland: An observational study
Source: PLoS Med. 2026 Nov 2;23(11):e1005288. doi: 10.1371/journal.pmed.1005288 (PMC13759143; doi:10.1371/journal.pmed.1005288)
Supplement: Supplementary file 17 [file pmed.1005288.s019.docx]

Suzanne De Bruijn, PhD	September 25, 2026

Associate Editor

PLOS Medicine

We have now carefully revised our manuscript entitled **"Trends in the Incidence of Childhood Type 1 Diabetes After the COVID-19 Lockdown in Finland: An Observational Study"** originally submitted to PLOS Medicine on March 6, 2026.

We highly appreciate the comments that we received from the Editor and the reviewer and have made corrections accordingly.

We agreed with most of the review comments and have now responded to these. However, if the reviewer and/or the editor appear unsatisfied with our response, we are willing to consider making further corrections.

GENERAL

* Please confirm that your title complies with PLOS Medicine's style. Your title must be nondeclarative and not a question. It should begin with main concept if possible. "Effect of" should be used only if causality can be inferred, i.e., for an RCT. Please place the study design ("A randomized controlled trial," "A retrospective study," "A modelling study," etc.) in the subtitle (ie, after a colon).

***Response:*** *We confirm that the manuscript title complies with PLOS Medicine’s style: “Trends in the Incidence of Childhood Type 1 Diabetes After the COVID-19 Lockdown in Finland: An Observational Study.”*

* Please confirm that your abstract complies with our requirements, including format (three sections: Background, Methods and Findings, and Conclusions) and providing all the information relevant to this study type <https://journals.plos.org/plosmedicine/s/submission-guidelines#loc->
abstract

***Response:*** *We confirm that our abstract complies with the journal's requirements and is structured using the mandatory headings: Background, Methods and Findings, and Conclusions.*

* In the last sentence of the Abstract Methods and Findings section, please describe the main limitation(s) of the study's methodology. We appreciate that you mention the short follow-up time, but please provide at least one other limitation of the study.

***Response:*** *In the final sentence of the Abstract's Methods and Findings section, we have explicitly stated the primary limitations of our methodology as follows: “Study limitations include a relatively short follow-up period after the lockdown and the lack of explicit modeling of the underlying long-term secular trend in type 1 diabetes incidence.”*

* Please ensure that the Introduction ends with a clear description of the study question or hypothesis.

***Response:*** *The Introduction section now concludes with the following explicit statement of the study hypothesis: “Based on other European studies, we expected that the lockdown measures* ***would be*** *associated with a higher incidence of type 1 diabetes. Consequently, we hypothesized that the disease rates after the lockdown would return to levels close to* ***those*** *observed before the lockdown started in Finland.”*

* Please ensure that all abbreviations are defined at first use throughout the text.

***Response:*** *We have ensured that all abbreviations are explicitly defined at their first use throughout the revised manuscript.*

* Please confirm that all numbers presented in the abstract are present and identical to numbers presented in the main manuscript text.

***Response:*** *We have carefully verified that all numerical data reported in the abstract are fully consistent with and identical to the numbers presented in the main text of the manuscript.*

* Please include a statement regarding use or non-use of AI tools and technologies at the end of the Methods, under the subheading: Use of AI Disclosure. Please specify the name(s) and version(s) of any tool(s) used and a description of their use, including how the AI-assisted or generated content was evaluated by authors, and a clear statement of which aspects of the study, article contents, data, or supporting files were affected or generated by AI tool usage. Please also include a statement specifying who retains responsibility for the final manuscript. See our guidelines for more information: <https://journals.plos.org/plosmedicine/s/ethical-publishing-practice#loc-artificial-intelligence-tools-and-technologies>.

***Response:*** *We have added a statement regarding usage of AI tools and technologies at the end of the Methods, under the subheading: Use of AI Disclosure as follows: “Use of AI DisclosureA Large Language Model (LLM) assistant (ChatGPT/Claude architecture, September 2026 version) was utilized during the revision stage of this manuscript. The tool was used exclusively for language editing, text polishing, and improving the stylistic flow and conciseness of the narrative text. Every suggestion provided by the AI tool was thoroughly evaluated, critically reviewed, and verified by the authors to ensure scientific accuracy and contextual alignment with the study. The usage of this tool was restricted strictly to text editing; no study concepts, primary hypotheses, raw datasets, statistical models, figure data, or supporting materials were generated, altered, or affected by the AI tool. The authors retain sole and absolute responsibility for the content, integrity, and final outputs of the manuscript.”*

* Statistical reporting: Please revise throughout the manuscript, including tables and figures.
- Please report statistical information as follows to improve clarity for the reader ""22% (95% CI [13,28]; p</=)"".

***Response:*** *We have thoroughly revised the manuscript, including all tables and figures, to report statistical information exactly in accordance with the requested format.*

- Please separate upper and lower bounds with commas instead of hyphens as the latter can be confused with reporting of negative values.

***Response:*** *We have revised all confidence intervals throughout the manuscript, tables, and figures to separate the upper and lower bounds with commas instead of hyphens, ensuring compliance with the journal's formatting guidelines.*

- Please repeat statistical definitions (HR, CI etc.) for each set of parentheses.

***Response:*** *We have revised the text to ensure that statistical definitions (such as IRR and CI) are consistently repeated for each set of parentheses throughout the manuscript.*

* The information on page 5 ('what's known' and 'what does this study add') is not necessary. Please remove.

***Response:*** *We have removed the information on page 5 accordingly.*

* Thank you for including the STROBE checklist. Please amend to use section and paragraph numbers, rather than page numbers. Please also provide this file as a separate supplement, rather than as part of the supplementary materials.

***Response:*** *We have updated the STROBE checklist to reference specific section and paragraph numbers instead of page numbers, ensuring it remains accurate even if text shifts during production. We have separated the STROBE checklist from the main supplementary materials file. It is now uploaded as an independent, standalone supplement file as requested.*

FUNDING STATEMENT
* Thank you for including your funding statement. We note that this is very long, and seems duplicated in the metadata. Please consider whether all these funders indeed provided support for this specific study. Please amend and make more concise if possible.

***Response:*** *Due to a technical limitation in the online submission system during the initial stages, we have been unable to modify the metadata fields directly. Following consultation with the journal office, we are providing the complete and corrected funding statement below. We kindly request that the editorial team updates the manuscript metadata accordingly:*

***FUNDING:*** *This study was supported by Helsinki University Hospital Research Funds (grant numbers TYH 2021316 and TYH2021343 to MK) [https://www.hus.fi], EU Horizon 2020 (Versatile emerging infectious disease observatory and Long Covids´project, grant number 874735 to OV) [https://europa.eu], Academy of Finland (grant numbers 350455, 308613 to MK and 351040 to OV) [https://www.aka.fi/en/], EU HERA: DURABLE (grant number 101102733 to OV) [https://europa.eu], Jane & Aatos Erkko Foundation (grant number 20242044 to OV) [https://jaes.fi/en/frontpage/], VTR (State funding for university-level health research) (grant number TYH2025391 to OV) [https://stm.fi], Sigrid Jusélius Foundation (un-numbered to OV and MK) [https://www.sigridjuselius.fi/en/], Medicinska understödsföreningen Liv och Hälsa (un-numbered to MK) [https://www.livochhalsa.fi/], University of Helsinki Doctoral School (UHDS) (un-numbered to ST) [https://www.helsinki.fi/en/research/doctoral-school], the Foundation for Pediatric Research in Finland (un-numbered to ST) [https://www.lastentautientutkimussaatio.fi/in-english/], and the Björkqvist Foundation (un-numbered to ST) [https://www.kangasala.fi/].*

*AUTHOR SUMMARY:*

** Under 'why was this research done' please consider adding an additional point, to provide more background to a layman's' audience.*

***Response:*** *An additional contextual point has been added to the lay summary to provide broader background information for a non-specialist audience:*

- *The Coronavirus disease 2019 (COVID-19) pandemic has been linked to an increased incidence of type 1 diabetes in numerous study reports.*
- *A few European studies have reported that the surge in type 1 diabetes incidence observed during the 2021 lockdowns did not persist after the reopening of society in 2022. However, studies investigating type 1 diabetes incidence beyond the acute phase of the pandemic remain scarce.*

** Under 'what did the researchers do and find' please provide a bit more explanation in the first bullet point. Please also consider switching point 2 and 3, to provide the data in a chronological order.*

***Response:*** *Following the Editor’s suggestion, more explanation has been added* *to the first bullet point to explicitly outline the time periods compared.*

*Regarding the order of the findings, we respectfully chose to retain the original sequence of the second and third bullet points. Because the primary objective of this specific analysis was to evaluate the immediate shift in type 1 diabetes incidence after the lockdown compared directly to the period during the lockdown, presenting these two highly related findings sequentially ensures the primary conclusion remains immediately accessible to the reader:*

- *The incidence of type 1 diabetes after the lockdown (09/2021–12/2022) was compared to the incidence during (03/2020–08/2021) and before lockdown (three 18-month terms 2014–2019) in Finnish children under the age of 15 years.*
- *The incidence of type 1 diabetes decreased by 15% among Finnish children during the first 16 months after the COVID-19 lockdown compared with the time when lockdown was implemented.*
- *The incidence after the lockdown was equal compared to the reference time before the COVID-19 pandemic.*

* In the author summary, in the final bullet point of 'What Do These Findings Mean?', please include the main limitations of the study in non-technical language.

***Response:*** *The primary methodological limitations have been incorporated into the text as follows: “Study limitations include a relatively short follow-up period after the lockdown and the lack of explicit modeling of the underlying long-term secular trend in type 1 diabetes incidence.”*

DATA AVAILABILITY
* PLOS Medicine requires that the de-identified data underlying the specific results in a published article be made available, without restrictions on access, in a public repository or as Supporting Information at the time of article publication, provided it is legal and ethical to do so. Please see the policy at <http://journals.plos.org/plosmedicine/s/data-availability> and FAQs at <http://journals.plos.org/plosmedicine/s/data-availability#loc-faqs-for-data-policy>

Please provide reasoning why the data cannot be shared, and what requirements researchers need to adhere to obtain access to the data from the Finnish pediatric diabetes registry and the Finndata.

***Response:*** *The de-identified, aggregated dataset underlying our primary outcomes—comparing the incidence rates of type 1 diabetes before, during, and after the COVID-19 lockdown period in Finland—has been made publicly available without restriction in a Zenodo repository README file. However, the individual-level datasets containing sensitive clinical characteristics, metabolic decompensation variables at diagnosis, islet autoimmunity markers, HLA genotypes, and combined SARS-CoV-2 exposure variables (antibody and vaccination status) cannot be publicly shared. Dissemination of these individual-level data is strictly prohibited under the European Union General Data Protection Regulation (GDPR) and the Finnish Act on the Secondary Use of Health and Social Data (552/2019) to protect patient privacy and eliminate the risk of re-identification.*

* We do not accept the statement "The de-identified aggregated data that underlie the results reported in this Article can be obtained from the corresponding author (mikael.knip@helsinki.fi) upon reasonable request."  Please provide this information, or provide a statement why this cannot be publicly available, and how researchers could get access to this information. Please note that the contact in that case cannot be a study author.

***Response:*** *Please find our fully updated and compliant Data Availability Statement below:*

***Data Availability Statement***

*The aggregated dataset required to replicate the primary incidence outcomes of this study has been deposited without restrictions in the Zenodo repository (*[*https://doi.org/10.5281/zenodo.22936130*](https://doi.org/10.5281/zenodo.22936130)*).*

*The individual-level clinical, genetic, and immunologic datasets underlying other reported variables cannot be made publicly available due to strict legal and ethical restrictions under Finnish national law and the European Union General Data Protection Regulation (GDPR). Under the Finnish Act on the Secondary Use of Health and Social Data (552/2019), public dissemination or direct author-sharing of individual-level or pseudonymized registry records is strictly prohibited to prevent patient re-identification.*

*These restricted clinical datasets are accessible to qualified researchers for replication purposes upon obtaining the required regulatory permits. To obtain access to the underlying data, researchers must adhere to the following access procedures:*

***Data Permit Request:*** *A formal data permit application must be submitted directly to* [*Findata (The Finnish Social and Health Data Permit Authority)*](https://www.google.com/goto?url=CAESTgHrOzAV6v4M353YPSTH7F6-BxgZD0_9KyLkTRTQ5Z1R7MTPZLnKEiIeZn3RWg0ZgJDX2uC63-8SvsOf7rM2YBsFq4Bpd_rAGJjgnBsKEg) *via the official Findata Permit Application Portal.*

***Analysis Environment:*** *In accordance with Findata infrastructure mandates, raw individual data cannot be downloaded locally. Approved researchers will be granted remote access to analyze the data within Findata’s secure computing environment (Kapseli).*

***Institutional Contact:*** *To verify data access criteria or request institutional assistance regarding the Finnish Pediatric Diabetes Register dataset, researchers may contact the independent data administration office at the University of Helsinki Faculty of Medicine (via email: med-ext-info@helsinki.fi or the Research Programs Unit administration at rpu-admin@helsinki.fi).*

* Please include the statement on code availability in the data availability statement. Please see guidance here:https://journals.plos.org/plosmedicine/s/code-sharing-guidance#loc-data-availability-statement-examples
* Please note that we do require code to be made available, and this cannot be upon request. Please provide the code as a supplement, or provide a URL to where the code can be found (e.g. Github, with a Zenodo DOI).

***Response:*** *We have integrated our code availability information into the final manuscript files and the master submission form as follows:*

***Code Availability:*** *All author-generated analytical code used to process the primary incidence data and secondary clinical traits have been made publicly available. The complete replication code repository is hosted on GitHub at* [*https://zenodo.org/records/22936130*](https://zenodo.org/records/22936130) *and has been permanently archived on Zenodo (https://doi.org/10.5281/zenodo.22936130).* 

TEXTUAL COMMENTS:

Intro:
-line 145: please remove ‘a series of research articles. This either needs references, or more detail, as you provide in the following sentences.

***Response:*** *In accordance with the Editor's suggestion, we have replaced the vague phrasing with a specific reference to a recent large-scale meta-analysis. This study combined data from 17 studies and demonstrated a systematic, global increase in childhood type 1 diabetes incidence during the acute phase of the pandemic.*

***Reference:*** *D’Souza D, Empringham J, Pechlivanoglou P, Uleryk EM, Cohen E, Shulman R, et al. Incidence of diabetes in children and adolescents during the COVID-19 pandemic: A systematic review and meta-analysis. JAMA Netw Open. 2023;6(8):e2321281.*

-line 152: include ‘incidence’. Also consider adding more detail, e.g something like: “it is unknown whether type 1 diabetes incidence after the pandemic returned to pre-pandemic levels”.

***Response:*** *In accordance with the reviewer's recommendation, we have updated the sentence to incorporate the word "incidence" and clarify the knowledge gap as follows: “However, studies investigating the incidence of type 1 diabetes beyond the acute phase of the pandemic remain scarce.”*

Methods:
225: please consider adding a definition for ‘strict’ and ‘loose’.

***Response:*** *In accordance with the reviewer's suggestion, we have clarified this sentence and directed the reader to our detailed definitions as follows: “To estimate the proportion of children diagnosed after the lockdown who had an antibody profile compatible with previous infection, we applied two distinct criteria: strict and loose (definitions for these criteria are provided in S1 Table).”*

FIGURES
* Please consider avoiding the use of red and green in order to make your figure more accessible. This also applies to supplementary figures.

***Response:*** *In accordance with the journal's accessibility guidelines, we have revised our figures to ensure they are fully color-blind friendly. Figures 1 and 2 do not utilize a red-green combination, and Supplementary Figures S4 and S5 have been completely modified to employ accessible color palettes. The updated supplementary file has been uploaded accordingly.*

*Figure S4:*

*Figure S5:*

* Figure 1: please clarify what the blue and white colours of the bars signify are in the legend.

***Response:*** *The figure legend for Figure 1 has been updated to explicitly define the bar colors and clarify the dual y-axes as follows:*

***FIGURE 1*** *Incidence rates of type 1 diabetes and prevalence of infection-induced SARS-CoV-2 antibodies across study periods. The graph displays data stratified by the reference period before the COVID-19 pandemic (three 18-month blocks spanning 2014–2019), during the societal lockdown (March 2020–August 2021), and after the lockdown (September 2021–December 2022). Blue bars represent the incidence rate of type 1 diabetes (expressed as new cases per 100,000 person-years in children aged <15 years) corresponding to the left y-axis. White bars represent the prevalence (%) of infection-induced SARS-CoV-2 antibodies among children newly diagnosed with type 1 diabetes corresponding to the right y-axis. Exact estimates and 95% confidence intervals (CIs) are shown above each bar. SARS-CoV-2 antibody status was not analysed for the pre-pandemic reference cohort.*

* Figure 2: please clarify what the colours used in the figure mean in the legend.

***Response:*** *The figure legend for Figure 2 has been updated to explicitly define the colors as follows:*

***FIGURE 2*** *Incidence rate ratios of type 1 diabetes stratified by sex and age group across study periods. The forest plot displays incidence rate ratios (IRRs) with their corresponding 95% confidence intervals (CIs) for Finnish children aged <15 years. Blue markers and horizontal bars represent the comparison between the pre-pandemic reference period (PreP: three 18-month blocks, 2014–2019) and the post-lockdown period (PostP: September 2021–December 2022). Red markers and horizontal bars represent the comparison between the societal lockdown period (LockD: March 2020–August 2021) and the post-lockdown period (PostP). The vertical dashed line indicates an IRR of 1.00 (no difference in incidence rates). Estimates are provided for the entire cohort ("All") and stratified by sex (Females, Males) across three specific age categories (<5 years, 5 to <10 years, and 10 to <15 years).*

Comments from Reviewers:

Reviewer #2: In this final resubmission, the authors discuss prior critiques clarifying the study features and claimed originalty / impact and made some amendment to the text.
Two remaining limitations should be made more explicit.
i) The analyses compare predefined calendar periods but do not model the underlying secular trend in childhood T1D incidence. Therefore, the similarity between post-lockdown and pre-pandemic incidence does not necessarily establish a return to the expected trajectory and cannot fully distinguish a transient increase from temporal displacement/compensation superimposed on the background trend.

***Response:*** *We thank the reviewer for this insightful methodological point. We agree that without explicit modeling of the long-term secular trend, we cannot definitively conclude that the post-lockdown incidence represents a perfect return to the expected historical trajectory, nor can we entirely disentangle temporal displacement from a transient surge. We have amended our manuscript to address this important nuance as follows:*

***Author summary:*** *“Study limitations include a relatively short follow-up period after the lockdown and the lack of explicit modeling of the underlying long-term secular trend in type 1 diabetes incidence.”*

***Abstract - Methods and Findings:*** *“Study limitations include a relatively short follow-up period after the lockdown and the lack of explicit modeling of the underlying long-term secular trend in type 1 diabetes incidence.”*

***Abstract - Conclusions:*** *“The lockdown represents a peak in childhood type 1 diabetes incidence in Finland compared to both the pre-pandemic and post-lockdown periods. However, the reversion toward historical levels after the lockdown does not necessarily establish a return to the expected historical trajectory, nor can it fully distinguish a transient change from temporal compensation superimposed on the background secular trend. The contrasting patterns between type 1 diabetes incidence and SARS-CoV-2 antibody prevalence during and after the lockdown suggest an indirect rather than a direct role of the virus in triggering the observed changes in incidence.”*

***Discussion – Study limitations:*** “*Study limitations include a relatively short follow-up period after the lockdown and the lack of explicit modeling of the underlying long-term secular trend in type 1 diabetes incidence.”*

***Discussion – Conclusions:*** *“Our study shows that a wave in the incidence of type 1 diabetes in children was observed after the onset of the COVID-19 pandemic. This included a peak in the incidence during the lockdown followed by a subsequent decline after the opening of the society. However, this reversion toward historical levels does not necessarily establish a return to the expected historical trajectory, nor can it fully distinguish a transient increase from temporal compensation superimposed on the background secular trend.”*

ii) The proposed indirect mechanisms, including reduced exposure to other infections, should be more clearly framed as speculative.

***Response:*** *We completely agree with the reviewer. While the contrasting patterns of T1D incidence and SARS-CoV-2 antibody prevalence point toward an indirect pathway, the exact underlying mechanisms—such as the "hygiene hypothesis" or reduced exposure to other seasonal viral infections during lockdown—remain speculative and cannot be directly proven by our data.*

*We have revised the Discussion section to ensure that these indirect pathways are explicitly framed as speculative mechanisms as follows:*

***Discussion: paragraph 5:*** *“While the opposing trends of type 1 diabetes incidence and SARS-CoV-2 antibody prevalence suggest an indirect pathway, the exact underlying mechanisms remain speculative. Factors such as the hygiene hypothesis or reduced lockdown exposure to other seasonal viruses cannot be directly proven by our register data.”*
