# Supplementary material for: Trends in the incidence of childhood type 1 diabetes after the COVID-19 lockdown in Finland: An observational study
Source: PLoS Med. 2026 Nov 2;23(11):e1005288. doi: 10.1371/journal.pmed.1005288 (PMC13759143; doi:10.1371/journal.pmed.1005288)
Supplement: Supplementary file 15 [file pmed.1005288.s017.docx]

Suzanne De Bruijn, PhD	June 23, 2026

Associate Editor

PLOS Medicine

We have now carefully revised our manuscript entitled **" Type 1 Diabetes and SARS-CoV-2 in Finnish Children After the COVID-19 Lockdown"** submitted to PLOS Medicine on March 6, 2026.

We highly appreciate the comments that we received from the reviewers and have made a series of corrections accordingly.

We agreed with most of the review comments and have now responded to these. However, if the reviewers and/or the editor appear unsatisfied with our response, we are willing to consider making further corrections.

Reviewer #1: This study compared the incidence of type 1 diabetes (T1D) before, during, and after the Covid-19 pandemic. The study's main strength is the paediatric diabetes register covering most of the Finnish population which is largely free from selection bias. The research question is interesting and timely. I have the following comments on the methods and interpretation:

1. Poisson regression was used to model incidence rate ratio. It is good to see the offset if properly incorporated but also also specify the outcome was the count. Please also specify the unit of analysis, is it by population subgroup? if so how were they defined? Also did the authors examine overinflation in the Poisson model?

**Response:** *We used Poisson regression analysis to model T1D incidence in Finnish children, using logged person-years (annual population size) as an offset to calculate incidence rate ratios across demographic and temporal subgroups (age, sex, and pandemic timeline). Counts of T1D cases served as a dependent variable.* *We used individual-level follow-up data (date of birth, date of T1D diagnosis) on T1D cases. Individual-level data on T1D cases were aggregated by age groups, sex and pandemic timeline similar to the PY data. Annual population statistics obtained from Statistics Finland (https://stat.fi/en/surveys) were used to determine person-years in each age and sex group (see manuscript page 11 line 217).*

*The study utilizes well-established nationwide registry data covering the entire pediatric population of Finland. When analyzing large, homogenous cohorts at a national level, the variance of the event counts naturally stabilizes and stays close to the mean. In such cases, standard Poisson models are considered robust enough to provide valid estimates, meaning any minor overdispersion would have a negligible impact on the calculated Incidence Rate Ratios (IRRs). Overdispersion would make the model's assumed standard errors too small, leading to artificially narrow confidence intervals and overly large statistical significances.* *We have applied robust standard errors calculated using the sandwich method (the Huber-White estimator) as a solution. The method corrects the variance-covariance matrix so that it is robust to structural deviations in the model (such as overdispersion or heteroscedasticity).*

2. Please specify whether age and sex have been accounted for in the model.

**Response:** *We did adjust our main analyses for age and sex. This has been mentioned in the statistical methods section of the manuscript (page 10, line 230). In addition, we separated the study population into three age categories: <5 years, 5 to <10 years, and 10 to <15 years.*

3. I also wondered if it would be a more revealing way to analyse the data using an interrupted time series, treating Covid pandemic as an 'intervention'. This way you could also control for long term trend, or change in long term trend if there's any. While I understand the number of cases are small, if possible showing more refined time-depending trend in finer resolution (e.g. via a spline? ) could provide more insight into what might be driving the trend. the current categorised presentation is a bit simplistic.

**Response:**
*Given that the COVID-19 pandemic acted as a diffuse, multi-phased disruption rather than a discrete single-point intervention, the current categorized presentation remains the most robust, transparent, and clinically interpretable approach for this sample size.*

*ITS analysis is best suited when an underlying secular trend is distinctive and undergoes a sudden and prominent change at a clear time point (e.g. due to an intervention) resulting either from the change in the level or the gradient of the trend, or both of these. The prerequisite is therefore both the existence of an observable and unambiguous trend before the "intervention", a common “intervention” point for all, after which a change in the trend can be expected to occur, and the strength of the effect sufficient to be detectable given the number of time points and counts or other units of observations per time point. Moreover, there are other known factors affecting power such as variability within the data and presence of time-varying confounding (Lopez Bernal et al., 2017). In the case of T1D incidence in Finland, there is a known non-homogeneity in the secular trend due to the age- and sex-specific variability and seasonality. Moreover, an essential step of the appropriate ITS analyses is proposing the impact model a priori, among others making beforehand assumptions about timing of the change (immediate, gradual, lagged change). Given these considerations, the results from the ITS can be threatened by a low power and data-driven (not subject-driven) approach to model selection.*

*In a recent study by Lehner and colleagues, with comparable number of T1D cases, ITS analyses were only able to show a change in the incidence level but not in the gradient. This "change in level" in their result is the same as what we have arrived at with the simplest analyses. Statistically establishing a change in the trend curve requires stronger data (the strength of the previous trend and the possible change in it in relation to the sample size to cancel out uncertainty). Based on these considerations, we conclude that additional analyses are unlikely to produce any valuable new information in our case.*

*References:*

*Lopez Bernal J, Cummins S, Gasparrini A. Interrupted time series regression for the evaluation of public health interventions: a tutorial. Int J Epidemiol. 2017;46(1):348–355.*

*Lehner CT, Anastasova I, Schauberger G, Eberl M, Schederecker F, Tauscher M, et al. Increasing incidence of type 1 diabetes in children and adolescents from 2012 to 2021 in Germany: trends before and during the COVID-19 pandemic. Eur J Epidemiol. 2026;2:1-2.*

4. Might be a bit speculative and outside the scope of the study, but it might be interesting to look at interaction of population level Covid cases and antibody level as a covariate in the above model i suggested, with the assumption that pre-covid pandemic there were 0 for both and these are ecological variable just acting as a rough proxy for how likely the T1D patients had these.

**Response:** *As the reviewer is suggesting we do think that it is too speculative and outside the scope of the study to look at interaction of population level COVID-19 cases and antibody level as a covariate when modeling the incidence of T1D.* *This conclusion is driven mainly by issues of data reliability, statistical power, and structural confounding. Most children with COVID-19 showed no symptoms, leading to massive undercounting in public case registries. Using population-level case rates to infer individual-level T1D risk introduces severe geographical and demographic biases. Requiring perfect antibody data would drastically shrink the available pool of subjects included in the study and weaken the statistical power of the analyses.*

5. The way the results are being reported is slightly confusing. I'd suggest to frame it chronologically: incidence higher during covid pandemic and than declined back to similar to prior covid.

***Response:*** *The 18-month long lockdown was used as the focal point in our previous article where we showed that the incidence of T1D increased during the lockdown compared to a period before lockdown (Knip et al. 2023). Therefore, we cannot use lockdown as the focal point in this paper. Since the reviewer finds our setup challenging, we have now clarified the text in the Abstract and the Discussion accordingly:*

*Conclusions of the Abstract:* ***“****The lockdown represents a peak in type 1 diabetes incidence in Finland, not only compared to the time before lockdown, but also afterwards.” (page 6 line 114)*

*The first paragraph of the Discussion: “The lockdown represents a peak in type 1 diabetes incidence in Finland, not only compared to the time before lockdown, but also afterwards.” (page 14, line 301)*

*Reference: Knip M, Parviainen A, Turtinen M****,*** *But A, Härkönen T, Hepojoki J,* *et al. SARS-CoV-2 and type 1 diabetes in children in Finland: an observational study. Lancet Diabetes Endocrinol. 2023;11(4):251-260.*

6. Abstract Conclusion - "Environmental changes can rapidly modify the incidence of type 1 diabetes and exogenous factors play a crucial role in the manifestation of clinical disease." this line reads out of place and not directly as a result of the findings. Suggest to remove.

***Response:*** *Our interpretation is that the present report suggests that environmental changes can rapidly modify the incidence of type 1 diabetes and exogenous factors play a crucial role in the manifestation of clinical disease. However, we agree that this is not the main conclusion and does not directly result from the findings. Therefore, we have removed this from the Abstract as proposed by the reviewer.*

Reviewer #2: This study used the Finnish registry to compare pediatric T1D incidence in the post-lockdown period to the lockdown period and antecedent periods. Results are interesting and novel.
General comment: I do not think that the main finding of the study is that the incidence of T1D "decreased" after lockdown. I would rather say that it returned to pre-lockdown levels. I would suggest this be also reflected in the title.

***Response:*** *We have now revised our manuscript and emphasized that the T1D incidences did not decline but returned to pre-lockdown levels.*

*Conclusions of the Abstract:* ***“****The lockdown represents a peak in type 1 diabetes incidence in Finland, not only compared to the time before lockdown, but also afterwards.” (page 6, line 114)*

*The first paragraph of the Discussion: “The lockdown represents a peak in type 1 diabetes incidence in Finland, not only compared to the time before lockdown, but also afterwards.” (page 14, line 301)*

*As PLOS Medicine has strict, specific guidelines for article titles we have not included the result in the title contrary to the reviewer’s comment. PLOS Medicine generally requires titles to be non-declarative and descriptive of the study's scope or focus, rather than stating the final result.*

-       As this is a retrospective study, I do not understand how it is possible that "To determine metabolic decompensation, blood pH and concentrations …". This part is described as it was standardized in a prospective study, which is not the case. Later: "A physician assessed the level of consciousness…" reads like a protocol / trial. The methods description should better align with study design.

***Response:*** *Many large-scale registers, such as the Finnish Pediatric Diabetes Register, routinely collect acute metabolic markers at the time of clinical presentation of a disease (T1D in our case).* *A cross-sectional design, such as in the present study, captures all these measurements at a single point in time. Consequently, it can only measure associations and establish a snapshot of metabolic status. Thereby, it cannot determine the exact temporal sequence that led to a particular state of decompensation. Following the reviewer comment we have now clarified the methods section so that it should be more evident for the readers that this is a register study and data was not prospectively collected.*

*Methods section “Analyses of clinical variables, autoantibodies and HLA genotypes” was revised as follows: “To determine metabolic decompensation, blood pH and concentrations of plasma β-hydroxybutyrate, glucose, and hemoglobin A_1c_ (HbA1c) were analyzed from blood samples collected from children with newly diagnosed type 1 diabetes. A physician assessed the level of consciousness (normal, altered, and unconscious) for each subject at the time of type 1 diabetes diagnosis. The duration of symptoms associated with type 1 diabetes before diagnosis was assessed through a questionnaire given to the family of each patient. Diabetic ketoacidosis (DKA) was defined as a blood pH < 7.30 in combination with ketonemia. Severe DKA was defined as a blood pH < 7.10.” (page 9, line 175)*

-       Why only IgG anti-SARS-CoV-2 were measured? Why not IgM? And what about swab analysis for active infection?

***Response:*** *In SARS-CoV-2 infections IgM antibodies, unlike in some other viral infections, have been reported not to be a particularly robust or sensitive marker for early infection and these antibodies also wane rapidly (Pecoraro et al, 2021, Semmler et al, 2021). Apart for IgG, we actually did measure also IgA class antibodies to SARS-CoV-2 spike protein, but this did not add to the detection of seropositive individuals beyond using IgG as a marker. In our study setting, we used IgG measurements to spike and nucleocapsid protein (the latter waning more rapidly with the assay used) to characterize and understand past infections and seroprevalence (how widespread the disease is in a population). Furthermore, the neutralizing antibody measurements actually comprise also all other antibody classes beyond IgG contributing to neutralization.*

*Only serum samples taken after the diagnosis of T1D were available for this study. Taking samples from children and diagnosing or registering the predominatly mild SARS-CoV-2 cases was anyhow uncommon in health care. We had no access to respiratory swabs or such results.*

*References:*

*Pecoraro V, Cassetti T, Meacci M,* [*Gargiulo*](https://pubmed.ncbi.nlm.nih.gov/?sort=date&size=20&term=Gargiulo+R&cauthor_id=33839972) *R, [Capobianchi](https://pubmed.ncbi.nlm.nih.gov/?sort=date&size=20&term=Capobianchi+MR&cauthor_id=33839972) MR,* [*Mussini*](https://pubmed.ncbi.nlm.nih.gov/?sort=date&size=20&term=Mussini+C&cauthor_id=33839972) *C, et al. Accuracy of the serological detection of IgG and IgM to SARS-Cov-2: a prospective, cross-sectional study. Eur J Clin Microbiol Infect Dis. 2021 Sep;40(9):1891-1898.*

*Semmler G, Traugott MT, Graninger M, Hoepler W, Seitz T, Kelani H, et al. Assessment of S1-, S2-, and NCP-Specific IgM, IgA, and IgG Antibody Kinetics in Acute SARS-CoV-2 Infection by a Microarray and Twelve Other Immunoassays. J Clin Microbiol. 2021;59(4):e02890-20.*

-       It seems that the registry forming the data source does not normally capture 100% of national cases of T1D. Therefore, is it possible that the variation in observed T1D incidence (relative 15%) is, at least in part, a reflection of better recording during one period compared to another? Is there any way to measure this or to rule it out? May this be a study limitation?

***Response:*** *In general terms, fluctuating reporting efficiency is a recognized methodological challenge in epidemiological studies. Variation in T1D incidence may be influenced by changes in data collection or registry completeness. However, validated studies consistently show that the FPDR captures more than 90% (specifically around 92%) of all newly diagnosed cases of pediatric diabetes (aged less than 15 years) across Finland. FPDR is widely considered exceptionally complete and high in coverage. However, there is no way to rule out the possibility that there were differences in recording of T1D cases during the different study periods as proposed by the reviewer. This has now been added as a limitation in the manuscript as follows:*

*“FPDR is widely considered exceptionally complete and high in coverage. However, there is no way to rule out the possibility that there were differences in recording of type 1 diabetes cases during the different study periods.” (page 17, line 370)*

-       That the incidence of T1D was truly reduced in males (0.82) but not in females (HR 0.90) does not seem to be supported by the data shown. Results are directionally consistent in the two sexes and, unless a formal interaction analysis (sex by period), this conclusion should be deleted.

***Response:*** *The reviewer is completely right to challenge this distinction, as concluding a true biological difference between sexes based on these numbers would be a statistical error. The IRRs for males (0.82) and females (0.90) are directionally aligned, both indicating a potential reduction in risk. Most importantly, the 95% CIs of the two groups overlap and that indicates that the association did not differ between the two sexes. We have now revised the manuscript accordingly.*

*The results section of the manuscript was revised as follows: We found evidence of a reduced type 1 diabetes incidence in males (IRR=0.82, 95% Cl 0.72 to 0.95, P=.007) but the decline in the incidence in females did not reach significance (IRR=0.90, 95% Cl .76 to 1.05, P=.185) (Fig. 1). (page 11, line 239)*

-       Please comment on the clinical significance of a <0.5 year difference in age, although statistical significant.

***Response:*** *The 95% confidence intervals (CI) for all three study periods (pre-pandemic, lockdown, post lockdown) heavily overlap with one another. The minor upward trend in the point estimates is likely due to random statistical variation rather than a true shifting epidemiological trend. In the context of pediatric biology and endocrinology, a fractional difference of 0.2 to 0.4 years (roughly 2.5 to 5 months) at this stage of life holds no clinical meaning. All three study cohorts are experiencing peak clinical onset during late mid-childhood, immediately preceding the physiological onset of adrenarche and gonadal puberty.*

-       Interpretation. Based on the data presented, it is hard to conclude that SARS-CoV-2 or COVID-19 per se were inducing T1D onset. Thus, it remains possible that the measures associated with lockdown (lifestyle, isolation, hygiene, and attention to health status [supported by clinical features at presentation]) transiently increased the incidence. Yet, the lockdown period was too short to influence the autoimmune process. Therefore, one may assume that the greater incidence during lockdown reflected, at least in part, an anticipated diagnosis in children who would probably have developed T1D anyway. Yet, in this case, the incidence rate in the subsequent period should be compensatory lower compared to the before-lockdown period (assuming no true change in T1D epidemiology in the period), which was not observed. Please comment on this.

-	Nonetheless, this phenomenon may be inscribed on the progressively raising T1D incidence in the majority of western countries (but not Finland, why?) since the last 10-15 years. In this sense, it would probably make more sense to perform sort of a regression analysis to understand whether the lockdown period represented a true deviation (peak) from the trend before and after. Then the incidence rate in the post-lockdown period, may turn out to be lower than expected based on the "secular" trend.

**Response:** *The study periods in our report are relatively short, with the pandemic interval and the aggregated pre-pandemic reference periods each covering 18 months and the post-pandemic period 16 months. We did detect some significant differences between study cohorts in autoantibodies; GADA positivity peaked in Finnish children diagnosed with type 1 diabetes during the COVID-19 pandemic lockdown period (as compared with pre-lockdown and postlockdown), although direct SARS-CoV-2 infection was unlikely the cause. Even so, a short follow-up study period should be seen as a limitation. This issue has been mentioned in the Discussion of the manuscript as follows:*

*“A short follow-up study period can be seen as a limitation.” (page 16, line 366)*

*The latter reviewer comment “the incidence rate in the subsequent period should be compensatory lower compared to the before-lockdown period (assuming no true change in T1D epidemiology in the period), which was not observed” is based on an expectation that the incidence of T1D does not change linearly as a function of time in relation to the lockdown measures. We argue that the follow-up period after the lockdown should have been twice as long as the current one, to detect the potential equal decrease in the incidence compared to the initial level, (i.e. pre-pandemic periods). If the incidence increased by 16% during the 18 months of the lockdown, we could, indeed, expect the incidence to drop by the same 16% during the next 18 months or so after the opening of society. Using this logic (linear change in the incidence in time), we could expect that after the postlockdown period, the incidence of T1D would continue to decrease for the next 16-18 months, and after that the incidence would start to rise again, reaching the baseline level after the next 16-18 months, i.e. the level before the lockdown. However, this remains to be investigated in future studies. See our free hand illustration below.*

*Given that the COVID-19 pandemic acted as a diffuse, multi-phased disruption rather than a discrete single-point intervention, the current categorized presentation remains the most robust, transparent, and clinically interpretable approach for this sample size.*

*ITS analysis is best suited when an underlying secular trend is distinctive and undergoes a sudden and prominent change at a clear time point (e.g. due to an intervention) resulting either from the change in the level or the gradient of the trend, or both of these. The prerequisite is therefore both the existence of an observable and unambiguous trend before the "intervention", a common “intervention” point for all, after which a change in the trend can be expected to occur, and the strength of the effect sufficient to be detectable given the number of time points and counts or other units of observations per time point. Moreover, there are other known factors affecting power such as variability within the data and presence of time-varying confounding (Lopez Bernal et al., 2017). In the case of T1D incidence in Finland, there is a known non-homogeneity in the secular trend due to the age- and sex-specific variability and seasonality. Moreover, an essential step of the appropriate ITS analyses is proposing the impact model a priori, among others making beforehand assumptions about timing of the change (immediate, gradual, lagged change). Given these considerations, the results from the ITS can be threatened by a low power and data-driven (not subject-driven) approach to model selection.*

*In a recent study by Lehner and colleagues, with comparable number of T1D cases, ITS analyses were only able to show a change in the incidence level but not in the gradient. This "change in level" in their result is the same as what we have arrived at with the simplest analyses. Statistically establishing a change in the trend curve requires stronger data (the strength of the previous trend and the possible change in it in relation to the sample size to cancel out uncertainty). Based on these considerations, we conclude that additional analyses are unlikely to produce any valuable new information in our case.*

*References:*

*Lopez Bernal J, Cummins S, Gasparrini A. Interrupted time series regression for the evaluation of public health interventions: a tutorial. Int J Epidemiol. 2017;46(1):348–355.*

*Lehner CT, Anastasova I, Schauberger G, Eberl M, Schederecker F, Tauscher M, et al.Gerlach R, Klug SJ. Increasing incidence of type 1 diabetes in children and adolescents from 2012 to 2021 in Germany: trends before and during the COVID-19 pandemic. Eur J Epidemiol. 2026;2:1-2.*

-       In the Discussion, the authors are invited to suggest what the lockdown experience can tell us on the occult mechanisms that may drive T1D onset, as a lesson learned, not limited to future lockdowns but more generally.

***Response:*** *The global pandemic lockdowns provided a rare, large-scale natural experiment that dramatically isolated human populations, offering groundbreaking insights into the poorly understood environmental triggers driving T1D onset. It is obvious that a substantial spike in pediatric new-onset T1D occurred during and immediately following the pandemic era in different countries including Finland. However, as the pandemic period was related to many different changes in children’s lives it is impossible to point out the exact triggers and mechanisms by which the increase in incidence took place. Therefore, based on the current research, we are hesitant to make more general conclusions about the causative agents triggering T1D. What the present report does, however, indicate is that environmental changes can rapidly modify the incidence of type 1 diabetes and exogenous factors play a crucial role in the manifestation of clinical disease.*

Reviewer #3: I enjoyed reading this clear and methodologically sound analysis of type 1 diabetes incidence and SARS-CoV-2 infection during different periods related to the COVID-19 pandemic in Finnish children. The result is interesting and of clinical and scientific interest.

Major comments:

It was difficult to determine what population had been included in the analysis of SARS-CoV-2 infection status. I have expanded further below, but overall I think it would be helpful to include a table or flowchart for both 'during lockdown' and 'post lockdown' cohorts to show the number of children tested for SARS-CoV-2 antibodies, their vaccination status and their classification according to both the 'strict' and 'loose' criteria. If there are a significant proportion with missing or unclear status, this should be discussed as a limitation in the Discussion

***Response:*** *We have now added flowcharts for the ”lockdown” and “post lockdown” periods as proposed by the reviewer. There was a significant proportion of unclear SARS-CoV-2 status, this was added as a limitation in the Discussion as proposed by the reviewer:*

*“However, distinguishing between antibodies from natural infection (anti-nucleocapsid) and vaccination (anti-spike) is logistically challenging and resulted in some cases with ambiguous infection status.”*

**S1 Figure.** Serum sample collection and SARS-CoV-2 antibody status in type 1 diabetes cases diagnosed during the lockdown in Finland.

**S2 Figure.** Serum sample collection and SARS-CoV-2 antibody status in type 1 diabetes cases diagnosed after the lockdown (09/2021–12/2022) in Finland. Only S positive samples were further tested for neutralizing antibodies against the variants they may have exposed to (considering the timing of the sampling and the time when each variant was circulating). Most samples that were positive in the neutralization test were positive against multiple variants.

**S3 Figure.** SARS-CoV-2 infection status of type 1 diabetes cases diagnosed during (03/2020–08/2021) and after the lockdown (09/2021–12/2022) in Finland. Definition of each criteria for the after lockdown cohort is explained in supplementary table 1.

From Figure 2 it appears the analysis of SARS-CoV-2 infection status was done on both the 'during lockdown' (03/2020-08/2021) and 'post lockdown' (09/2021-12/2022) cohorts. The Results text reads '489 index cases (83.8%)' but I'm not sure what this is 83.8% of. The Results text then references Figure 2, but I'm not sure this links to the numbers in the text. It appears the text, and Supplement Figures 1 and 2 all refer to the post lockdown cohort only? Supplement Figure 2 is two pie charts - it would be useful to have the actual numbers of patients too (either change pie chart to a table or add numbers to pie chart).

***Response:*** *The 489 index cases (83.8%) refer to subjects that were diagnosed after the lockdown. This has now been clarified in the results section accordingly (page13, line 276). The corresponding proportion of cases for the lockdown period was 0.9%. This information has been previously published in our Lancet Diabetes and Endocrinology article (reference). As the reviewer is suggesting the Supplemental Figures 1 and 2 (in the revised version Supplementary Figures 4 and 5) refer to the post lockdown cohort only. We have clarified the text and the figure captions accordingly. Following the reviewer recommendation we have now added the actual numbers of subjects in the pie chart Supplement Figure 4.*

*Reference: Knip M, Parviainen A, Turtinen M, But A, Härkönen T, Hepojoki J,* *et al. SARS-CoV-2 and type 1 diabetes in children in Finland: an observational study. Lancet Diabetes Endocrinol. 2023;11(4):251-260.*

**S4 Figure.** SARS-CoV-2 infection and vaccination status in Finnish children diagnosed with type 1 diabetes after the lockdown (09/2021–12/2022). The status is classified as ‘infection’ (has had SARS-CoV-2 infection at some point in the past), ‘no infection’ (no evidence of SARS-CoV-2 infection in the past), or ‘ambiguous’ (could not be determined). Percentages (numbers of observations). Two different classification criteria (strict and loose, see S1 Table) were used, both leading to same overall conclusions. Children who had received vaccination less than 14 days before sampling (n = 4, unclear whether the vaccine had yet had time to inducean antibody response) are not included.

*The results section was revised as follows:*

*“SARS-CoV-2 antibodies were analyzed in 489 index cases diagnosed after the lockdown (83·8%). Of these, 317 (64·8%) tested positive for SARS-CoV-2 spike (S) antibodies by an enzyme-linked immunosorbent assay (ELISA), out of whom 303 also scored positive (titer ≥20) for neutralizing antibodies (Fig 2).”*

Minor comments:

*       Missing full stop at the end of the Methods

***Response:*** *We have added a full stop at the end of the Methods.*

*       X-axis label for Figure 2 has misspelt 'Tyype'

***Response:*** *We have corrected spelling of the word “type” in Figure 2.*

*       Sentence in Results 'The prevalence of SARS-CoV-2 antibodies and vaccination rates in Finnish children diagnosed with type 1 diabetes in the postlockdown between September 1, 2021 and December 31, 2022…'  appears to be missing a verb.

***Response:*** *We have deleted this sentence.*

*       I think it would be helpful to the reader to have the time periods for the pre/during/post lockdown cohorts included in the Figure and Table captions.

***Response:*** *We have added the time periods for the three cohorts in the figure and table captions.*

Please upload any figures associated with your paper as individual TIF or EPS files with 300dpi resolution at resubmission; please read our figure guidelines for more information on our requirements: <http://journals.plos.org/plosmedicine/s/figures>. While revising your submission, we strongly recommend that you use PLOS's NAAS tool (<https://ngplosjournals.pagemajik.ai/artanalysis>) to test your figure files. NAAS can convert your figure files to the TIFF file type and meet basic requirements (such as print size, resolution), or provide you with a report on issues that do not meet our requirements and that NAAS cannot fix.

After uploading your figures to PLOS's NAAS tool - <https://ngplosjournals.pagemajik.ai/artanalysis>, NAAS will process the files provided and display the results in the "Uploaded Files" section of the page as the processing is complete.

If the uploaded figures meet our requirements (or NAAS is able to fix the files to meet our requirements), the figure will be marked as "fixed" above. If NAAS is unable to fix the files, a red "failed" label will appear above.

When NAAS has confirmed that the figure files meet our requirements, please download the file via the download option, and include these NAAS processed figure files when submitting your revised manuscript.

***Response:*** *We have uploaded figures as EPS or TIF files with 300 dpi resolution.*

*	We expect all researchers with submissions to PLOS in which author-generated code underpins the findings in the manuscript to make all author-generated code available without restrictions upon publication of the work. In cases where code is central to the manuscript, we require the code to be made available as a condition of publication. Authors are responsible for ensuring that the code is reusable and well documented. Please make any custom code available, either as part of your data deposition or as a supplementary file. Please add a sentence to your data availability statement regarding any code used in the study, e.g. "The code used in the analysis is available from Github [URL] and archived in Zenodo [DOI link]" Please review our guidelines at <https://journals.plos.org/plosmedicine/s/materials-software-and-code-sharing> and ensure that your code is shared in a way that follows best practice and facilitates reproducibility and reuse. Because Github depositions can be readily changed or deleted, we encourage you to make a permanent DOI'd copy (e.g. in Zenodo) and provide the URL.

***Response:*** *We have modified our data availability agreement as follows: “*DATA AVAILABILITY STATEMENT: Codes for the statistical analyses are available upon request. Aggregated data related to type 1 diabetes incidences may be obtained via an application from the Finnish Pediatric Diabetes Register

Email: [pedia-diabetesrekisteri@helsinki.fi](mailto:pedia-diabetesrekisteri@helsinki.fi)

Access to COVID-19 vaccination data may be obtained by sending an application to Findata Information website: <https://findata.fi/en/>

Email: [info@findata.fi](mailto:info@findata.fi)“

*	Please disclose any use of AI tools and technologies to the study or article content in the Methods, including the name(s) of any tool(s) used and a description of their use. Please see our guidelines for more information: <https://journals.plos.org/plosmedicine/s/ethical-publishing-practice#loc-artificial-intelligence-tools-and-technologies>.

***Response:*** *No AI tools or technologies were used in this study.*

*Financial statement: Thank you for providing a thorough statement.

a) Please include grant numbers for all grants, or clarify in the rebuttal that grant numbers did not exist.

***Response:*** We have now added grant numbers for all grants or clarified in the rebuttal those grants for which numbers did not exist as follows:

*“FUNDING: This study was supported by Helsinki University Hospital Research Funds (grant numbers TYH 2021316 and TYH2021343), EU Horizon 2020 (Versatile emerging infectious disease observatory and Long Covids´project,  grant number 874735), Academy of Finland, (grant numbers 350455, 351040 and 308613), EU HERA: DURABLE 101102733, JAES 20242044, VTR (State funding for university-level health research) TYH2025391, Sigrid Jusélius Foundation (un-numbered), Jane & Aatos Erkko Foundation (un-numbered), Medicinska understödsföreningen Liv och Hälsa (un-numbered), University of Helsinki Doctoral School (UHDS) (un-numbered), the Foundation for Pediatric Research in Finland and the Björkqvist Foundation (un-numbered).”*

b) Please consider removing the conflict of interest statement from this financial disclosure; This can go in the COI statement instead

***Response:*** The conflict of interest statement was removed from financial disclosure to COI statement.

c) Consider if the URLs can be included in brackets after mentioning the funder, instead of in a separate list afterwards.

***Response:*** Not applicable

d) Consider if the statement can be made more concise; it currently feels like all funders are mentioned 2 or 3 times.

***Response:*** All funders are mentioned only once.

* Data availability policy: PLOS Medicine requires that the de-identified data underlying the specific results in a published article be made available, without restrictions on access, in a public repository or as Supporting Information at the time of article publication, provided it is legal and ethical to do so. Please see the policy at <http://journals.plos.org/plosmedicine/s/data-availability> and FAQs at

<http://journals.plos.org/plosmedicine/s/data-availability#loc-faqs-for-data-policy>.

***Response:*** *We have modified our data availability agreement as follows: “Codes for the statistical analyses are available upon request. Aggregated data related to type 1 diabetes incidences may be obtained via an application from the Finnish Pediatric Diabetes Register*

*Email: pedia-diabetesrekisteri@helsinki.fi*

*Access to COVID-19 vaccination data may be obtained by sending an application to Findata Information website: https://findata.fi/en/*

*email: info@findata.fi “*

* Ethics statement: Thank you for including this. Please include the ethics committee approval number. Please also specify whether informed consent was oral or written (or waived, and why).

***Response:*** *We have now included the ethics committee approval number: THL/5342/6.02.04/202. The informed consent was written.*

FORMATTING - GENERAL

* Please ensure that your title complies with PLOS Medicine's style. Your title must be nondeclarative and not a question. It should begin with main concept if possible. "Effect of" should be used only if causality can be inferred, i.e., for an RCT. Please place the study design ("A randomized controlled trial," "A retrospective study," "A modelling study," etc.) in the subtitle (ie, after a colon).

***Response:*** *We have modified the title of our manuscript accordingly as follows: “Type 1 Diabetes and SARS-CoV-2 in Finnish Children After the COVID-19 Lockdown: An Observational Study”*

*	Abstract: Please structure your abstract using the PLOS Medicine headings (Background, Methods and Findings, Conclusions). Please combine the Methods and Findings sections into one section.

***Response:*** *We have corrected the abstract so that it includes PLOS Medicine headings*.

* In the last sentence of the Abstract Methods and Findings section, please describe the main limitation(s) of the study's methodology.

***Response:*** *We now describe the main limitation(s) as follows: “A short follow-up study period after the lockdown is a study limitation.” (page 6, line 113)*

*	At this stage, we ask that you include a short, non-technical Author Summary of your research to make findings accessible to a wide audience that includes both scientists and non-scientists. The Author Summary should immediately follow the Abstract in your revised manuscript. This text is subject to editorial change and should be distinct from the scientific abstract. Ideally each sub-heading should contain 2-3 single sentence, concise bullet points containing the most salient points from your study. In the final bullet point of 'What Do These Findings Mean?', please include the main limitations of the study in non-technical language. Please see our author guidelines for more information: <https://journals.plos.org/plosmedicine/s/revising-your-manuscript#loc-author-summary>.

***Response:*** *We have now added the following Author Summary (page 7, line 122):*

***Why was this study done?***

- *While COVID-19 lockdowns have been systematically associated with increased incidence rate of type 1 diabetes, less is known about the disease rates after lockdowns.*

***What did the researchers do and find?***

- *We examined the incidence of type 1 diabetes after the SARS-CoV-2 pandemic related lockdown in Finland.*
- *The incidence of type 1 diabetes decreased by 16% among Finnish children during the first 16 months after the COVID-19 lockdown.*
- *The rate of type 1 diabetes increased by an equal percentage during the initial 18-month lockdown period compared to the reference time before the COVID-19 pandemic.*

***What do these findings mean?***

- *The lockdown represents a peak in type 1 diabetes incidence in Finland, not only compared to the time before lockdown, but also afterwards.*
- *Further long-term cross-national investigations on the topic are warranted.*

*	Please express the main results with 95% CIs as well as p values. When reporting p values please report as p<0.001 and where higher as the exact p value p=0.002, for example. Throughout, we strongly suggest reporting statistical information as follows to improve clarity for the reader: "22% (95% CI [13%,28%]; p</=)". Please be sure to define all numerical values at first use.

***Response:*** *The reporting of the results was corrected throughout the manuscript.*

*	Please cite the reference numbers in square brackets. Citations should precede punctuation.

***Response:*** *The reference numbers were corrected throughout the manuscript.*

*	Please remove the 'conclusions' subheading from the discussion. Please also remove any other subheadings from the discussion.

***Response:*** *All subheadings were removed from the discussion accordingly.*

FIGURES AND TABLES
 *	Please define all abbreviations used in each figure/table (including those in Supporting Information files).

***Response:*** *All abbreviations used in each figure/table were defined.*

*	Please consider avoiding the use of red and green in order to make your figure more accessible to those with color blindness. Please also consider this for the supplementary files.

***Response:*** *We use red and dark blue/bright blue in our figures. We tested the figures with a red-green color-blind person to verify that the figures are accessible to those with color blindness.*

SUPPLEMENTARY MATERIAL
 *	Please note that supplementary material will be posted as supplied by the authors. Therefore, please amend it according to the relevant comments outlined here.

*	Please cite your Supporting Information as outlined here: <https://journals.plos.org/plosmedicine/s/supporting-information>

***Response:*** *We have corrected the citation of our Supporting Information accordingly (page 17, line 388).*
REFERENCES
 *	PLOS uses the numbered citation (citation-sequence) method and first six authors, et al.

***Response:*** *We have corrected the reference list accordingly.*

*	Please ensure that journal name abbreviations match those found in the National Center for Biotechnology Information (NCBI) databases (<http://www.ncbi.nlm.nih.gov/nlmcatalog/journals>), and are appropriately formatted and capitalised.

***Response:*** *We have verified that journal name abbreviations match those found in the NCBI databases*.

*	Where website addresses are cited, please include the complete URL and specify the date of access (e.g. [accessed: 12/06/2023]).

***Response:*** *We do not cite websites.*

*	Please also see https://journals.plos.org/plosmedicine/s/submission-guidelines#loc-references for further details on reference formatting.

***Response:*** *Additional author guidelines were followed.*

OBSERVATIONAL STUDIES

*	Abstract: Please include the study design, population and setting, number of participants, years during which the study took place (enrollment and follow up), length of follow up, and main outcome measures.

***Response:*** *We have now included the study design, population and setting, number of participants, years during which the study took place (enrollment and follow up), length of follow up, and main outcome measures in the abstract. (page 6, line 97)*

*	[FOR POPULATION HEALTH/REGISTRY STUDIES] Please ensure that the study is reported according to the RECORD guideline (available from [[https://www.record-statement.org)]https://www.record-statement.org](https://www.record-statement.org)]https/www.record-statement.org)) and include the completed checklist as Supporting Information. Please add the following statement, or similar, to the Methods: "This study is reported as per the Reporting of Studies Conducted using Observational Routinely-Collected Data (RECORD) guideline (S1 Checklist)." When completing the checklist, please use section and paragraph numbers, rather than page numbers.

***Response:*** *We have now included the STROBE checklist accordingly.*

*	For all observational studies, in the manuscript text, please indicate: (1) the specific hypotheses you intended to test, (2) the analytical methods by which you planned to test them, (3) the analyses you actually performed, and (4) when reported analyses differ from those that were planned, transparent explanations for differences that affect the reliability of the study's results. If a reported analysis was performed based on an interesting but unanticipated pattern in the data, please be clear that the analysis was data driven.

***Response:*** *The listed requirements are indicated in our manuscript.*

*	Please state in the Methods section whether the study had a prospective protocol or analysis plan. If a prospective analysis plan (from your funding proposal, IRB or other ethics committee submission, study protocol, or other planning document written before analyzing the data) was used in designing the study, please include the relevant document(s) with your revised manuscript as a Supporting Information file to be published alongside your study and cite it in the Methods section. A legend for this file should be included at the end of your manuscript. If no such document exists, please make sure that the Methods section transparently describes when analyses were planned, and when/why any data-driven changes to analyses took place. Changes in the analysis, including those made in response to peer review comments, should be identified as such in the Methods section of the paper, with rationale.

***Response:*** *The prospective analysis plan is included in the online only Supplement.*
