# Supplementary material for: Trends in the incidence of childhood type 1 diabetes after the COVID-19 lockdown in Finland: An observational study
Source: PLoS Med. 2026 Nov 2;23(11):e1005288. doi: 10.1371/journal.pmed.1005288 (PMC13759143; doi:10.1371/journal.pmed.1005288)
Supplement: Supplementary file 16 [file pmed.1005288.s018.docx]

Suzanne De Bruijn, PhD	August 17, 2026

Associate Editor

PLOS Medicine

We have now carefully revised our manuscript entitled **" Type 1 Diabetes and SARS-CoV-2 in Finnish Children After the COVID-19 Lockdown"** originally submitted to PLOS Medicine on March 6, 2026.

We highly appreciate the comments that we received from the reviewers and have made corrections accordingly.

We agreed with most of the review comments and have now responded to these. However, if the reviewers and/or the editor appear unsatisfied with our response, we are willing to consider making further corrections.

Reviewer #1: Thank you for the clarifications. I have no further comments.

***Response:*** *Thank you for your time and for the valuable feedback that helped improve the manuscript.*

Reviewer #2: Outstanding points.
- The current title "Type 1 Diabetes 1 and SARS-CoV-2 in Finnish Children After the COVID-19 Lockdown" is misleading because SARS-CoV-2 is not the focus of the study and the term incidence should be added.

***Response:*** *We agree with the reviewer’s suggestion. We have revised the title to explicitly include the word "incidence" and to specify the study design as per the journal's guidelines. The title has been modified as follows: “Trends in the Incidence of Childhood Type 1 Diabetes After the COVID-19 Lockdown in Finland: An Observational Study”*

- Overall, the study seems inconclusive, being unable to assess whether there were true changes in the incidence of T1D or a transient deviation that compensated after lockdown. The conclusion that "The lockdown represents a peak in type 1 diabetes incidence in Finland, not only compared to the time before lockdown, but also afterwards" does not seem to be very original. A compensatory decline after lockdown remains likely but cannot be supported by available data.
Considering the prior publications, this seems to be a minor advancement.

***Response:*** *We thank the reviewer for this constructive critique regarding the novelty and the temporal scope of our study. We welcome the opportunity to clarify the unique value, clinical relevance, and epidemiological context of our manuscript.*

*We agree that an extended post-lockdown follow-up period would provide further longitudinal insights. However, we maintain that our current 16-month follow-up period is methodologically rigorous, scientifically valid, and highly comparable to the rest of the study's design:*

- ***Temporal Balance:*** *The 16-month post-lockdown window closely mirrors the 18-month acute lockdown period and the 18-month historical reference periods. This balanced epoch design allows for direct, reliable statistical comparisons across all three distinct phases, minimising the confounding variables and statistical noise that often arise from vastly unequal observation windows.*
- ***Data Restrictions:*** *Furthermore, extending this cohort beyond this window is unfortunately not possible due to external systemic factors. Nationwide comprehensive monitoring by the Finnish Pediatric Diabetes Register had to be discontinued in 2023 due to severe national funding constraints. Consequently, our dataset captures the maximum available window of comprehensive national tracking for this cohort.*

***Regarding the novelty, originality, and clinical interest:*** *While we acknowledge that regional studies have separately tracked pediatric diabetes trends following the pandemic, our study offers a multi-dimensional epidemiological dataset that is entirely unique in the existing literature:*

- ***Triangulated Variable Analysis & Mechanistic Insights:*** *To our knowledge, this is the first study to directly synthesise and compare concurrent shifts across three critical concurrent vectors: nationwide T1D incidence rates accompanied with islet autoantibody status of the newly diagnosed cases, SARS-CoV-2 antibody seroprevalence data, and COVID-19 vaccination coverage. By cross-referencing real-time T1D onset trends directly against antibody status and vaccination rates, this dataset provides highly valuable insights into the role of the virus itself versus other environmental or immunising factors on incidence shifts. This level of triangulation offers rare epidemiological evidence to assess the likelihood of whether SARS-CoV-2 acts as a direct viral trigger for type 1 diabetes.*
- ***Complete Nationwide Coverage:*** *Unlike most of the existing literature relying on single-center or localised regional cohorts, our data encompasses the entire country of Finland. Because Finland holds the highest background incidence of T1D globally, nationwide data from this population provides highly rigorous, statistically powered insights crucial for global modelling.*

***Alignment with current research gaps and lack of consensus:*** *The clinical interest and urgency of our data are highlighted by recent high-level calls for evidence in the field. Notably, a comprehensive review article published in the September issue of Diabetologia (Said et al. Diabetologia 2026;69:2396–2407) explicitly demonstrates that there is currently no clear scientific consensus on whether COVID-19 directly causes T1D, confirming that this topic remains highly active and intensely debated.*

*The Diabetologia review highlights two major gaps that our study directly remedies:*

- ***Lack of Biochemical Confirmation of T1D:*** *The authors point out that only a small percentage of published pandemic-era papers pursued biochemical confirmation of autoimmunity of the newly diagnosed cases via systematic islet autoantibody measurements. Thereby, some of the T2D cases may have been mistakenly diagnosed as T1D across studies, thereby falsely adding to the observed T1D peaks in different countries. Our paper directly addresses this deficit by incorporating islet antibody (IAA, GADA, IA-2A and ZnT8A) status tracking alongside T1D incidences in the three study periods.*
- ***Unresolved Changes in the Incidences:*** *The review emphasises that substantial knowledge gaps remain regarding why T1D incidence rate peaks occurred across different countries during the acute pandemic, noting that high-quality, comprehensive datasets are urgently required to untangle these variances.*

*By providing a complete, triple-variable nationwide data of Finland that addresses the gaps in scientific knowledge identified by Diabetologia, our study directly moves this hot debate forward. This makes it highly relevant and impactful for the broad medical audience of PLOS Medicine.*

***We trust that these points clarify our methodological choices and the unique value of our dataset. However, should the reviewer still disagree with our argumentation, we would highly appreciate their detailed feedback accompanied by relevant literature or references, so that we may better understand their perspective and further strengthen the manuscript.***

***Reference:*** *Said N, Jones I, Anderson-Baucum EK, Evans-Molina C. SARS-CoV-2 and diabetes: a post-pandemic reappraisal. Diabetologia. 2026;69(9):2396-2407. doi:10.1007/s00125-026-06785-4*

Reviewer #3: I thank the authors for addressing all of my previous comments. My only comment is that the following sentence in the Discussion is perhaps a little too definitive: "In fact, such an increase or decrease has never been detected during the whole history of data collection." It might read better as: "To our knowledge, no increase or decrease of this magnitude has been observed in the available type 1 diabetes incidence data to date".

***Response:*** *We agree with the reviewer and thank them for pointing out this oversight. We recognise that the original phrasing was overly broad. We have now amended the sentence to clarify that this lack of fluctuation refers strictly to the history of the Finnish Pediatric Diabetes Register (FPDR): "In fact, such an increase or decrease has never been detected during the whole history of* ***FPDR*** *data collection."*
