# Supplementary material for: Trends in the incidence of childhood type 1 diabetes after the COVID-19 lockdown in Finland: An observational study
Source: PLoS Med. 2026 Nov 2;23(11):e1005288. doi: 10.1371/journal.pmed.1005288 (PMC13759143; doi:10.1371/journal.pmed.1005288)
Supplement: Peer review history

# Editor report: Decision Letter 0

**DOI:** 10.1371/journal.pmed.1005288.r001

**Authors:** Suzanne De Bruijn

10 Mar 2026

Dear Dr Tall,

Thank you for submitting your manuscript entitled "Type 1 Diabetes and SARS-CoV-2 in Finnish Children

After the COVID-19 Lockdown" for consideration by PLOS Medicine.

Your manuscript has now been evaluated by the PLOS Medicine editorial staff, and I am writing to let you know that we would like to send your submission out for external peer review.

However, before we can send your manuscript to reviewers, we need you to complete your submission by providing the metadata that is required for full assessment. To this end, please login to Editorial Manager where you will find the paper in the 'Submissions Needing Revisions' folder on your homepage. Please click 'Revise Submission' from the Action Links and complete all additional questions in the submission questionnaire.

For clinical studies, please upload a copy of your trial study protocol as a supporting information file. The study protocol should be the version submitted for approval to the institutional review board or ethics committee, should include any amendments to the study protocol, as well as the date of their approval by the institutional review or ethics committee. Please also detail any deviations from the study protocol in the Methods section of your manuscript. The editors will consider the protocol and study conduct prior to a final decision for external review.

Please re-submit your manuscript within two working days, i.e. by Mar 12 2026 11:59PM.

Login to Editorial Manager here: https://www.editorialmanager.com/pmedicine

Once your full submission is complete, your paper will undergo a series of checks in preparation for peer review. Once your manuscript has passed all checks it will be sent out for review.

Feel free to email us at plosmedicine@plos.org if you have any queries relating to your submission.

Kind regards,

Suzanne De Bruijn, PhD

Associate Editor

PLOS Medicine

# Peer review: Decision Letter 1

**DOI:** 10.1371/journal.pmed.1005288.r002

**Authors:** Suzanne De Bruijn

2 Jun 2026

Dear Dr Tall,

Many thanks for submitting your manuscript "Type 1 Diabetes and SARS-CoV-2 in Finnish Children

After the COVID-19 Lockdown" (PMEDICINE-D-26-00821R1) to PLOS Medicine. The paper has been reviewed by subject experts and a statistician; their comments are included below and can also be accessed here: [LINK]

As you will see, the reviewers thought your study is interesting and of clinical interest. However, there were also several comments, including on the analysis that was performed. After discussing the paper with the editorial team and an academic editor with relevant expertise, I'm pleased to invite you to revise the paper in response to the reviewers' comments. We would ask you to address all comments, and strongly encourage you to perform the additional analyses suggested by the reviewers. We plan to send the revised paper to some or all of the original reviewers, and we cannot provide any guarantees at this stage regarding publication.

I've also included some editorial requests, which you can find at the bottom of this email. Please include these in your point-by-point response.

In addition to these revisions, you may need to complete some formatting changes, which you will receive in a follow up email. A member of our team will be in touch with a set of requests shortly. If you do not receive a separate email within a few days, please assume that checks have been completed, and no additional changes are required.

When you upload your revision, please include a point-by-point response that addresses all of the reviewer and editorial points, indicating the changes made in the manuscript and either an excerpt of the revised text or the location (eg: page and line number) where each change can be found. Please also be sure to check the general editorial comments at the end of this letter and include these in your point-by-point response. When you resubmit your paper, please include a clean version of the paper as the main article file and a version with changes tracked as a marked-up manuscript. It may also be helpful to check the guidelines for revised papers at http://journals.plos.org/plosmedicine/s/revising-your-manuscript for any that apply to your paper.

We ask that you submit your revision by Jun 23 2026 11:59PM. However, if this deadline is not feasible, please contact me by email, and we can discuss a suitable alternative.

Don't hesitate to contact me directly with any questions (sbruijn@plos.org).

Best regards,

Suzanne

Suzanne De Bruijn, PhD

Associate Editor

PLOS Medicine

sbruijn@plos.org

-----------------------------------------------------------

Comments from the academic editor:

As suggested by the reviewers, I also find this interesting and of clinical significance.

I would strongly encourage the authors to provide more evidence based on the suggestions by the statistical reviewer.

-----------------------------------------------------------

Comments from the reviewers:

Reviewer #1: This study compared the incidence of type 1 diabetes (T1D) before, during, and after the Covid-19 pandemic. The study's main strength is the paediatric diabetes register covering most of the Finnish population which is largely free from selection bias. The research question is interesting and timely. I have the following comments on the methods and interpretation:

1. Poisson regression was used to model incidence rate ratio. It is good to see the offset if properly incorporated but also also specify the outcome was the count. Please also specify the unit of analysis, is it by population subgroup? if so how were they defined? Also did the authors examine overinflation in the Poisson model?

2. Please specify whether age and sex have been accounted for in the model.

3. I also wondered if it would be a more revealing way to analyse the data using an interrupted time series, treating Covid pandemic as an 'intervention'. This way you could also control for long term trend, or change in long term trend if there's any. While I understand the number of cases are small, if possible showing more refined time-depending trend in finer resolution (e.g. via a spline? ) could provide more insight into what might be driving the trend. the current categorised presentation is a bit simplistic.

4. Might be a bit speculative and outside the scope of the study, but it might be interesting to look at interaction of population level Covid cases and antibody level as a covariate in the above model i suggested, with the assumption that pre-covid pandemic there were 0 for both and these are ecological variable just acting as a rough proxy for how likely the T1D patients had these.

5. The way the results are being reported is slightly confusing. I'd suggest to frame it chronologically: incidence higher during covid pandemic and than declined back to similar to prior covid.

6. Abstract Conclusion - "Environmental changes can rapidly modify the104incidence of type 1 diabetes and exogenous factors play a crucial role in the manifestation of clinical disease." this line reads out of place and not directly as a result of the findings. Suggest to remove.

Reviewer #2: This study used the Finnish registry to compare pediatric T1D incidence in the post-lockdown period to the lockdown period and antecedent periods. Results are interesting and novel.

General comment: I do not think that the main finding of the study is that the incidence of T1D "decreased" after lockdown. I would rather say that it returned to pre-lockdown levels. I would suggest this be also reflected in the title.

-As this is a retrospective study, I do not understand how it is possible that "To determine metabolic decompensation, blood pH and concentrations …". This part is described as it was standardized in a prospective study, which is not the case. Later: "A physician assessed the level of consciousness…" reads like a protocol / trial. The methods description should better align with study design.

-Why only IgG anti-SARS-CoV-2 were measured? Why not IgM? And what about swab analysis for active infection?

-It seems that the registry forming the data source does not normally capture 100% of national cases of T1D. Therefore, is it possible that the variation in observed T1D incidence (relative 15%) is, at least in part, a reflection of better recording during one period compared to another? Is there any way to measure this or to rule it out? May this be a study limitation?

-That the incidence of T1D was truly reduced in males (0.82) but not in females (HR 0.90) does not seem to be supported by the data shown. Results are directionally consistent in the two sexes and, unless a formal interaction analysis (sex by period), this conclusion should be deleted.

-Please comment on the clinical significance of a <0.5 year difference in age, although statistical significant.

-Interpretation. Based on the data presented, it is hard to conclude that SARS-CoV-2 or COVID-19 per se were inducing T1D onset. Thus, it remains possible that the measures associated with lockdown (lifestyle, isolation, hygiene, and attention to health status [supported by clinical features at presentation]) transiently increased the incidence. Yet, the lockdown period was too short to influence the autoimmune process. Therefore, one may assume that the greater incidence during lockdown reflected, at least in part, an anticipated diagnosis in children who would probably have developed T1D anyway. Yet, in this case, the incidence rate in the subsequent period should be compensatory lower compared to the before-lockdown period (assuming no true change in T1D epidemiology in the period), which was not observed. Please comment on this.

-Nonetheless, this phenomenon may be inscribed on the progressively raising T1D incidence in the majority of western countries (but not Finland, why?) since the last 10-15 years. In this sense, it would probably make more sense to perform sort of a regression analysis to understand whether the lockdown period represented a true deviation (peak) from the trend before and after. Then the incidence rate in the post-lockdown period, may turn out to be lower than expected based on the "secular" trend.

-In the Discussion, the authors are invited to suggest what the lockdown experience can tell us on the occult mechanisms that may drive T1D onset, as a lesson learned, not limited to future lockdowns but more generally.

Reviewer #3: I enjoyed reading this clear and methodologically sound analysis of type 1 diabetes incidence and SARS-CoV-2 infection during different periods related to the COVID-19 pandemic in Finnish children. The result is interesting and of clinical and scientific interest.

Major comments:

It was difficult to determine what population had been included in the analysis of SARS-CoV-2 infection status. I have expanded further below, but overall I think it would be helpful to include a table or flowchart for both 'during lockdown' and 'post lockdown' cohorts to show the number of children tested for SARS-CoV-2 antibodies, their vaccination status and their classification according to both the 'strict' and 'loose' criteria. If there are a significant proportion with missing or unclear status, this should be discussed as a limitation in the Discussion.

From Figure 2 it appears the analysis of SARS-CoV-2 infection status was done on both the 'during lockdown' (03/2020-08/2021) and 'post lockdown' (09/2021-12/2022) cohorts. The Results text reads '489 index cases (83.8%)' but I'm not sure what this is 83.8% of. The Results text then references Figure 2, but I'm not sure this links to the numbers in the text. It appears the text, and Supplement Figures 1 and 2 all refer to the post lockdown cohort only? Supplement Figure 2 is two pie charts - it would be useful to have the actual numbers of patients too (either change pie chart to a table or add numbers to pie chart).

Minor comments:

*Missing full stop at the end of the Methods

*X-axis label for Figure 2 has misspelt 'Tyype'

*Sentence in Results 'The prevalence of SARS-CoV-2 antibodies and vaccination rates in Finnish children diagnosed with type 1 diabetes in the postlockdownbetween September 1, 2021 and December 31, 2022…' appears to be missing a verb.

*I think it would be helpful to the reader to have the time periods for the pre/during/post lockdown cohorts included in the Figure and Table captions.

Any attachments provided with reviews can be seen via the following link: [LINK]

--------------------------------------------------------- ---

General editorial requests:

*Please upload any figures associated with your paper as individual TIF or EPS files with 300dpi resolution at resubmission; please read our figure guidelines for more information on our requirements: http://journals.plos.org/plosmedicine/s/figures. While revising your submission, we strongly recommend that you use PLOS's NAAS tool (https://ngplosjournals.pagemajik.ai/artanalysis) to test your figure files. NAAS can convert your figure files to the TIFF file type and meet basic requirements (such as print size, resolution), or provide you with a report on issues that do not meet our requirements and that NAAS cannot fix.

After uploading your figures to PLOS's NAAS tool - https://ngplosjournals.pagemajik.ai/artanalysis, NAAS will process the files provided and display the results in the "Uploaded Files" section of the page as the processing is complete.

If the uploaded figures meet our requirements (or NAAS is able to fix the files to meet our requirements), the figure will be marked as "fixed" above. If NAAS is unable to fix the files, a red "failed" label will appear above.

When NAAS has confirmed that the figure files meet our requirements, please download the file via the download option, and include these NAAS processed figure files when submitting your revised manuscript.

*We expect all researchers with submissions to PLOS in which author-generated code underpins the findings in the manuscript to make all author-generated code available without restrictions upon publication of the work. In cases where code is central to the manuscript, we require the code to be made available as a condition of publication. Authors are responsible for ensuring that the code is reusable and well documented. Please make any custom code available, either as part of your data deposition or as a supplementary file. Please add a sentence to your data availability statement regarding any code used in the study, e.g. "The code used in the analysis is available from Github [URL] and archived in Zenodo [DOI link]" Please review our guidelines at https://journals.plos.org/plosmedicine/s/materials-software-and-code-sharing and ensure that your code is shared in a way that follows best practice and facilitates reproducibility and reuse. Because Github depositions can be readily changed or deleted, we encourage you to make a permanent DOI'd copy (e.g. in Zenodo) and provide the URL.

* Please disclose any use of AI tools and technologies to the study or article content in the Methods, including the name(s) of any tool(s) used and a description of their use. Please see our guidelines for more information: https://journals.plos.org/plosmedicine/s/ethical-publishing-practice#loc-artificial-intelligence-tools-and-technologies.

*Financial statement: Thank you for providing a thorough statement.

a) Please include grant numbers for all grants, or clarify in the rebuttal that grant numbers did not exist.

b) Please consider removing the conflict of interest statement from this financial disclosure; This can go in the COI statement instead

c) Consider if the URLs can be included in brackets after mentioning the funder, instead of in a separate list afterwards.

d) Consider if the statement can be made more concise; it currently feels like all funders are mentioned 2 or 3 times.

* Data availability policy: PLOS Medicine requires that the de-identified data underlying the specific results in a published article be made available, without restrictions on access, in a public repository or as Supporting Information at the time of article publication, provided it is legal and ethical to do so. Please see the policy at http://journals.plos.org/plosmedicine/s/data-availability and FAQs at

http://journals.plos.org/plosmedicine/s/data-availability#loc-faqs-for-data-policy.

* Ethics statement: Thank you for including this. Please include the ethics committee approval number. Please also specify whether informed consent was oral or written (or waived, and why).

FORMATTING - GENERAL

* Please ensure that your title complies with PLOS Medicine's style. Your title must be nondeclarative and not a question. It should begin with main concept if possible. "Effect of" should be used only if causality can be inferred, i.e., for an RCT. Please place the study design ("A randomized controlled trial," "A retrospective study," "A modelling study," etc.) in the subtitle (ie, after a colon).

*Abstract: Please structure your abstract using the PLOS Medicine headings (Background, Methods and Findings, Conclusions). Please combine the Methods and Findings sections into one section.

* In the last sentence of the Abstract Methods and Findings section, please describe the main limitation(s) of the study's methodology.

*At this stage, we ask that you include a short, non-technical Author Summary of your research to make findings accessible to a wide audience that includes both scientists and non-scientists. The Author Summary should immediately follow the Abstract in your revised manuscript. This text is subject to editorial change and should be distinct from the scientific abstract. Ideally each sub-heading should contain 2-3 single sentence, concise bullet points containing the most salient points from your study. In the final bullet point of 'What Do These Findings Mean?', please include the main limitations of the study in non-technical language. Please see our author guidelines for more information: https://journals.plos.org/plosmedicine/s/revising-your-manuscript#loc-author-summary.

*Please express the main results with 95% CIs as well as p values. When reporting p values please report as p<0.001 and where higher as the exact p value p=0.002, for example. Throughout, we strongly suggest reporting statistical information as follows to improve clarity for the reader: "22% (95% CI [13%,28%]; p</=)". Please be sure to define all numerical values at first use.

*Please cite the reference numbers in square brackets. Citations should precede punctuation.

* Please remove the 'conclusions' subheading from the discussion. Please also remove any other subheadings from the discussion.

FIGURES AND TABLES

*Please define all abbreviations used in each figure/table (including those in Supporting Information files).

*Please consider avoiding the use of red and green in order to make your figure more accessible to those with color blindness. Please also consider this for the supplementary files.

SUPPLEMENTARY MATERIAL

*Please note that supplementary material will be posted as supplied by the authors. Therefore, please amend it according to the relevant comments outlined here.

*Please cite your Supporting Information as outlined here: https://journals.plos.org/plosmedicine/s/supporting-information

REFERENCES

*PLOS uses the numbered citation (citation-sequence) method and first six authors, et al.

*Please ensure that journal name abbreviations match those found in the National Center for Biotechnology Information (NCBI) databases (http://www.ncbi.nlm.nih.gov/nlmcatalog/journals), and are appropriately formatted and capitalised.

*Where website addresses are cited, please include the complete URL and specify the date of access (e.g. [accessed: 12/06/2023]).

*Please also see https://journals.plos.org/plosmedicine/s/submission-guidelines#loc-references for further details on reference formatting.

OBSERVATIONAL STUDIES

*Abstract: Please include the study design, population and setting, number of participants, years during which the study took place (enrollment and follow up), length of follow up, and main outcome measures.

*[FOR POPULATION HEALTH/REGISTRY STUDIES] Please ensure that the study is reported according to the RECORD guideline (available from https://www.record-statement.org) and include the completed checklist as Supporting Information. Please add the following statement, or similar, to the Methods: "This study is reported as per the Reporting of Studies Conducted using Observational Routinely-Collected Data (RECORD) guideline (S1 Checklist)." When completing the checklist, please use section and paragraph numbers, rather than page numbers.

*For all observational studies, in the manuscript text, please indicate: (1) the specific hypotheses you intended to test, (2) the analytical methods by which you planned to test them, (3) the analyses you actually performed, and (4) when reported analyses differ from those that were planned, transparent explanations for differences that affect the reliability of the study's results. If a reported analysis was performed based on an interesting but unanticipated pattern in the data, please be clear that the analysis was data driven.

*Please state in the Methods section whether the study had a prospective protocol or analysis plan. If a prospective analysis plan (from your funding proposal, IRB or other ethics committee submission, study protocol, or other planning document written before analyzing the data) was used in designing the study, please include the relevant document(s) with your revised manuscript as a Supporting Information file to be published alongside your study and cite it in the Methods section. A legend for this file should be included at the end of your manuscript. If no such document exists, please make sure that the Methods section transparently describes when analyses were planned, and when/why any data-driven changes to analyses took place. Changes in the analysis, including those made in response to peer review comments, should be identified as such in the Methods section of the paper, with rationale.

# Author comment: Author response to Decision Letter 2

**DOI:** 10.1371/journal.pmed.1005288.r003

1 Jul 2026

Attachment Submitted filename: Review-kommentit_23.6.26.docx

# Peer review: Decision Letter 2

**DOI:** 10.1371/journal.pmed.1005288.r004

**Authors:** Suzanne De Bruijn

6 Aug 2026

Dear Dr Tall,

I am writing on behalf of my colleague Suzanne de Bruijn. Many thanks for submitting your manuscript "Type 1 Diabetes and SARS-CoV-2 in Finnish Children

After the COVID-19 Lockdown: An Observational Study" (PMEDICINE-D-26-00821R2) to PLOS Medicine. The paper has been reviewed by subject experts and a statistician; their comments are included below and can also be accessed here: [LINK]

As you will see, while reviewers 1 and 3 are largely satisfied with the revisions to the manuscript, reviewer 2 and the academic editor continue to raise outstanding concerns and do not support publication of the study at this time. In particular, reviewer 2 and the academic editor are not persuaded that the response to reviewer 2's comments raised in the last round of review are sufficient to provide the novel insights required for publication in PLOS Medicine. After discussing the paper with the editorial team and an academic editor with relevant expertise, we invite you to revise the paper in response to the reviewers' comments in one final round of review. Please note that with regard to the short follow-up, as the data exists to extend this analysis, we ask that you include it in your revised manuscript in order to address the concern raised. We plan to send the revised paper to some or all of the original reviewers, and we cannot provide any guarantees at this stage regarding publication. Please be advised that we may seek the input of an additional independent reviewer to consider at revised manuscript.

In addition to these revisions, you may need to complete some formatting changes, which you will receive in a follow up email. A member of our team will be in touch with a set of requests shortly. If you do not receive a separate email within a few days, please assume that checks have been completed, and no additional changes are required.

When you upload your revision, please include a point-by-point response that addresses all of the reviewer and editorial points, indicating the changes made in the manuscript and either an excerpt of the revised text or the location (eg: page and line number) where each change can be found. Please also be sure to check the general editorial comments at the end of this letter and include these in your point-by-point response. When you resubmit your paper, please include a clean version of the paper as the main article file and a version with changes tracked as a marked-up manuscript. It may also be helpful to check the guidelines for revised papers at http://journals.plos.org/plosmedicine/s/revising-your-manuscript for any that apply to your paper.

We ask that you submit your revision by Aug 27 2026 11:59PM. However, if this deadline is not feasible, please contact me by email, and we can discuss a suitable alternative.

Don't hesitate to contact me directly with any questions (sbruijn@plos.org).

Best regards,

Alison

Alison Farrell, PhD

Senior Editor

PLOS Medicine

sbruijn@plos.org

-----------------------------------------------------------

Paraphrased comments from the academic editor:

The authors' response to the Reviewer 2 didn't solve the key questions the reviewer raised in the last round of review. The authors should provide additional analyses to highlight the innovation and significance of this study, instead of proving something that has already been reported. Please address the suggestions of reviewer 2 in full and discuss further the issue of interpretation of the results.

-----------------------------------------------------------

Comments from the reviewers:

Reviewer #1: Thank you for the clarifications. I have no further comments.

Reviewer #2: Outstanding points.

- The current title "Type 1 Diabetes 1 and SARS-CoV-2 in Finnish Children After the COVID-19 Lockdown" is misleading because SARS-CoV-2 is not the focus of the study and the term incidence should be added.

- Overall, the study seems inconclusive, being unable to assess whether there were true changes in the incidence of T1D or a transient deviation that compensated after lockdown. The conclusion that "The lockdown represents a peak in type 1 diabetes incidence in Finland, not only compared to the time before lockdown, but also afterwards" does not seem to be very original. A compensatory decline after lockdown remains likely but cannot be supported by available data.

Considering the prior publications, this seems to be a minor advancement.

Reviewer #3: I thank the authors for addressing all of my previous comments. My only comment is that the following sentence in the Discussion is perhaps a little too definitive: "In fact, such an increase or decrease has never been detected during the whole history of data collection." It might read better as: "To our knowledge, no increase or decrease of this magnitude has been observed in the available type 1 diabetes incidence data to date".

Any attachments provided with reviews can be seen via the following link: [LINK]

--------------------------------------------------------- ---

General editorial requests:

(Note: not all will apply to your paper, but please check each item carefully)

*We ask every co-author listed on the manuscript to fill in a contributing author statement, making sure to declare all competing interests. If any of the co-authors have not filled in the statement, we will remind them to do so when the paper is revised. If all statements are not completed in a timely fashion this could hold up the re-review process. If new competing interests are declared later in the revision process, this may also hold up the submission. Should there be a problem getting one of your co-authors to fill in a statement we will be in contact. Please do not add or remove authors without first discussing this with the handling editor. You can see our competing interests policy here: http://journals.plos.org/plosmedicine/s/competing-interests.

*Please upload any figures associated with your paper as individual TIF or EPS files with 300dpi resolution at resubmission; please read our figure guidelines for more information on our requirements: http://journals.plos.org/plosmedicine/s/figures. While revising your submission, we strongly recommend that you use PLOS's NAAS tool (https://ngplosjournals.pagemajik.ai/artanalysis) to test your figure files. NAAS can convert your figure files to the TIFF file type and meet basic requirements (such as print size, resolution), or provide you with a report on issues that do not meet our requirements and that NAAS cannot fix.

After uploading your figures to PLOS's NAAS tool - https://ngplosjournals.pagemajik.ai/artanalysis, NAAS will process the files provided and display the results in the "Uploaded Files" section of the page as the processing is complete.

If the uploaded figures meet our requirements (or NAAS is able to fix the files to meet our requirements), the figure will be marked as "fixed" above. If NAAS is unable to fix the files, a red "failed" label will appear above.

When NAAS has confirmed that the figure files meet our requirements, please download the file via the download option, and include these NAAS processed figure files when submitting your revised manuscript.

*Please ensure that the paper adheres to the PLOS Data Availability Policy (see http://journals.plos.org/plosmedicine/s/data-availability), which requires that all data underlying the study's findings be provided in a repository or as Supporting Information. For data residing with a third party, authors are required to provide instructions with contact information (web or email address) for obtaining the data. Please note that a study author cannot be the contact person for the data. PLOS journals do not allow statements supported by "data not shown" or "unpublished results." For such statements, authors must provide supporting data or cite public sources that include it.

*We expect all researchers with submissions to PLOS in which author-generated code underpins the findings in the manuscript to make all author-generated code available without restrictions upon publication of the work. In cases where code is central to the manuscript, we require the code to be made available as a condition of publication. Authors are responsible for ensuring that the code is reusable and well documented. Please make any custom code available, either as part of your data deposition or as a supplementary file. Please add a sentence to your data availability statement regarding any code used in the study, e.g. "The code used in the analysis is available from Github [URL] and archived in Zenodo [DOI link]" Please review our guidelines at https://journals.plos.org/plosmedicine/s/materials-software-and-code-sharing and ensure that your code is shared in a way that follows best practice and facilitates reproducibility and reuse. Because Github depositions can be readily changed or deleted, we encourage you to make a permanent DOI'd copy (e.g. in Zenodo) and provide the URL.

*Please ensure that the study is reported according to the STROBE guideline and include the completed STROBE checklist as Supporting Information. When completing the checklist, please use section and paragraph numbers, rather than page numbers. Please add the following statement, or similar, to the Methods: "This study is reported as per STROBE guideline (S1 Checklist)."

Please include a disclosure regarding use or non-use of AI in generating the results and manuscript.

FORMATTING - GENERAL

*Abstract: Please structure your abstract using the PLOS Medicine headings (Background, Methods and Findings, Conclusions). Please combine the Methods and Findings sections into one section.

*At this stage, we ask that you include a short, non-technical Author Summary of your research to make findings accessible to a wide audience that includes both scientists and non-scientists. The Author Summary should immediately follow the Abstract in your revised manuscript. This text is subject to editorial change and should be distinct from the scientific abstract. Ideally each sub-heading should contain 2-3 single sentence, concise bullet points containing the most salient points from your study. In the final bullet point of 'What Do These Findings Mean?', please include the main limitations of the study in non-technical language. Please see our author guidelines for more information: https://journals.plos.org/plosmedicine/s/revising-your-manuscript#loc-author-summary.

*Please express the main results with 95% CIs as well as p values. When reporting p values please report as p<0.001 and where higher as the exact p value p=0.002, for example. Throughout, suggest reporting statistical information as follows to improve clarity for the reader "22% (95% CI [13%,28%]; p</=)". Please be sure to define all numerical values at first use.

*Please include page numbers and line numbers in the manuscript file. Use continuous line numbers (do not restart the numbering on each page).

*Please cite the reference numbers in square brackets. Citations should precede punctuation.

FIGURES AND TABLES

*Please provide titles and legends for all figures and tables (including those in Supporting Information files).

*Please define all abbreviations used in each figure/table (including those in Supporting Information files).

*Please consider avoiding the use of red and green in order to make your figure more accessible to those with color blindness.

SUPPLEMENTARY MATERIAL

*Please note that supplementary material will be posted as supplied by the authors. Therefore, please amend it according to the relevant comments outlined here.

*Please cite your Supporting Information as outlined here: https://journals.plos.org/plosmedicine/s/supporting-information

REFERENCES

*PLOS uses the numbered citation (citation-sequence) method and first six authors, et al.

*Please ensure that journal name abbreviations match those found in the National Center for Biotechnology Information (NCBI) databases (http://www.ncbi.nlm.nih.gov/nlmcatalog/journals), and are appropriately formatted and capitalised.

*Where website addresses are cited, please include the complete URL and specify the date of access (e.g. [accessed: 12/06/2023]).

*Please also see https://journals.plos.org/plosmedicine/s/submission-guidelines#loc-references for further details on reference formatting.

OBSERVATIONAL STUDIES

*Abstract: Please include the study design, population and setting, number of participants, years during which the study took place (enrollment and follow up), length of follow up, and main outcome measures.

*Please ensure that the study is reported according to the STROBE (or appropriate STOBE extension) guideline (available from: https://www.equator-network.org/reporting-guidelines/strobe) and include the completed STROBE (or STROBE extension) checklist as Supporting Information. Please add the following statement, or similar, to the Methods: "This study is reported as per the Strengthening the Reporting of Observational Studies in Epidemiology (STROBE) guideline (S1 Checklist)." When completing the checklist, please use section and paragraph numbers, rather than page numbers.

*[FOR POPULATION HEALTH/REGISTRY STUDIES] Please ensure that the study is reported according to the RECORD guideline (available from https://www.record-statement.org) and include the completed checklist as Supporting Information. Please add the following statement, or similar, to the Methods: "This study is reported as per the Reporting of Studies Conducted using Observational Routinely-Collected Data (RECORD) guideline (S1 Checklist)." When completing the checklist, please use section and paragraph numbers, rather than page numbers.

*[FOR POPULATION HEALTH ESTIMATES] Please ensure that the study is reported according to the GATHER statement (available from https://www.equator-network.org/reporting-guidelines/gather-statement) and include the completed checklist as Supporting Information. Please add the following statement, or similar, to the Methods: "This study is reported as per the Guidelines for Accurate and Transparent Health Estimates Reporting (GATHER) statement (S1 Checklist)." When completing the checklist, please use section and paragraph numbers, rather than page numbers.

*[FOR MEDIATION ANALYSES] We recommend that the study is reported according to the AGReMA statement (https://agrema-statement.org/#:~:text=AGReMA%20is%20an%20evidence%2D%20and,randomised%20trials%20and%20observational%20studies) and include the completed checklist as Supporting Information. Please add the following statement, or similar, to the Methods: "This study is reported as per the Guideline for Reporting Mediation Analyses (AGReMA) statement (S1 Checklist)." When completing the checklist, please use section and paragraph numbers, rather than page numbers.

*For all observational studies, in the manuscript text, please indicate: (1) the specific hypotheses you intended to test, (2) the analytical methods by which you planned to test them, (3) the analyses you actually performed, and (4) when reported analyses differ from those that were planned, transparent explanations for differences that affect the reliability of the study's results. If a reported analysis was performed based on an interesting but unanticipated pattern in the data, please be clear that the analysis was data driven.

*Please state in the Methods section whether the study had a prospective protocol or analysis plan. If a prospective analysis plan (from your funding proposal, IRB or other ethics committee submission, study protocol, or other planning document written before analyzing the data) was used in designing the study, please include the relevant document(s) with your revised manuscript as a Supporting Information file to be published alongside your study and cite it in the Methods section. A legend for this file should be included at the end of your manuscript. If no such document exists, please make sure that the Methods section transparently describes when analyses were planned, and when/why any data-driven changes to analyses took place. Changes in the analysis, including those made in response to peer review comments, should be identified as such in the Methods section of the paper, with rationale.

# Author comment: Author response to Decision Letter 3

**DOI:** 10.1371/journal.pmed.1005288.r005

18 Aug 2026

Attachment Submitted filename: Response to Review Comments 17.8.26.docx

# Peer review: Decision Letter 3

**DOI:** 10.1371/journal.pmed.1005288.r006

**Authors:** Suzanne De Bruijn

16 Sep 2026

Dear Dr. Tall,

Thank you very much for re-submitting your manuscript "Trends in the Incidence of Childhood Type 1 Diabetes After the COVID-19 Lockdown in Finland: An Observational Study" (PMEDICINE-D-26-00821R3) for review by PLOS Medicine.

I have discussed the paper with my colleagues and the academic editor and it was also seen again by one of the reviewers. I am pleased to say that provided the remaining reviewer comments, as well as editorial and production issues are dealt with we are planning to accept the paper for publication in the journal.

The remaining issues that need to be addressed are listed at the end of this email. Any accompanying reviewer attachments can be seen via the link below. Please take these into account before resubmitting your manuscript:

[LINK]

***Please note while forming your response, if your article is accepted, you may have the opportunity to make the peer review history publicly available. The record will include editor decision letters (with reviews) and your responses to reviewer comments. If eligible, we will contact you to opt in or out.***

In revising the manuscript for further consideration here, please ensure you address the specific points made by each reviewer and the editors. In your rebuttal letter you should indicate your response to the reviewers' and editors' comments and the changes you have made in the manuscript. Please submit a clean version of the paper as the main article file. A version with changes marked must also be uploaded as a marked up manuscript file.

Please also check the guidelines for revised papers at http://journals.plos.org/plosmedicine/s/revising-your-manuscript for any that apply to your paper. If you haven't already, we ask that you provide a short, non-technical Author Summary of your research to make findings accessible to a wide audience that includes both scientists and non-scientists. The Author Summary should immediately follow the Abstract in your revised manuscript. This text is subject to editorial change and should be distinct from the scientific abstract.

In addition to these revisions, you may need to complete some formatting changes, which you will receive in a follow up email. A member of our team will be in touch with a set of requests shortly. If you do not receive a separate email within a few days, please assume that checks have been completed, and no additional changes are required.

We expect to receive your revised manuscript within 1 week. Please email us (plosmedicine@plos.org) if you have any questions or concerns.

We ask every co-author listed on the manuscript to fill in a contributing author statement. If any of the co-authors have not filled in the statement, we will remind them to do so when the paper is revised. If all statements are not completed in a timely fashion this could hold up the re-review process. Should there be a problem getting one of your co-authors to fill in a statement we will be in contact. YOU MUST NOT ADD OR REMOVE AUTHORS UNLESS YOU HAVE ALERTED THE EDITOR HANDLING THE MANUSCRIPT TO THE CHANGE AND THEY SPECIFICALLY HAVE AGREED TO IT.

Please review your reference list to ensure that it is complete and correct. If you have cited papers that have been retracted, please include the rationale for doing so in the manuscript text, or remove these references and replace them with relevant current references. Any changes to the reference list should be mentioned in the rebuttal letter that accompanies your revised manuscript.

Please note, when your manuscript is accepted, an uncorrected proof of your manuscript will be published online ahead of the final version, unless you've already opted out via the online submission form. If, for any reason, you do not want an earlier version of your manuscript published online or are unsure if you have already indicated as such, please let the journal staff know immediately at plosmedicine@plos.org.

If you have any questions in the meantime, please contact me or the journal staff on plosmedicine@plos.org.

We look forward to receiving the revised manuscript by Sep 23 2026 11:59PM.

Sincerely,

Suzanne De Bruijn, PhD

Associate Editor

PLOS Medicine

plosmedicine.org

------------------------------------------------------------

Requests from Editors:

GENERAL

* Please confirm that your title complies with PLOS Medicine's style. Your title must be nondeclarative and not a question. It should begin with main concept if possible. "Effect of" should be used only if causality can be inferred, i.e., for an RCT. Please place the study design ("A randomized controlled trial," "A retrospective study," "A modelling study," etc.) in the subtitle (ie, after a colon).

* Please confirm that your abstract complies with our requirements, including format (three sections: Background, Methods and Findings, and Conclusions) and providing all the information relevant to this study type https://journals.plos.org/plosmedicine/s/submission-guidelines#loc-

abstract

* In the last sentence of the Abstract Methods and Findings section, please describe the main limitation(s) of the study's methodology. We appreciate that you mention the short follow-up time, but please provide at least one other limitation of the study.

* Please ensure that the Introduction ends with a clear description of the study question or hypothesis.

* Please ensure that all abbreviations are defined at first use throughout the text.

* Please confirm that all numbers presented in the abstract are present and identical to numbers presented in the main manuscript text.

* Please include a statement regarding use or non-use of AI tools and technologies at the end of the Methods, under the subheading: Use of AI Disclosure. Please specify the name(s) and version(s) of any tool(s) used and a description of their use, including how the AI-assisted or generated content was evaluated by authors, and a clear statement of which aspects of the study, article contents, data, or supporting files were affected or generated by AI tool usage. Please also include a statement specifying who retains responsibility for the final manuscript. See our guidelines for more information: https://journals.plos.org/plosmedicine/s/ethical-publishing-practice#loc-artificial-intelligence-tools-and-technologies.

* Statistical reporting: Please revise throughout the manuscript, including tables and figures.

- Please report statistical information as follows to improve clarity for the reader ""22% (95% CI [13,28]; p</=)"".

- Please separate upper and lower bounds with commas instead of hyphens as the latter can be confused with reporting of negative values.

- Please repeat statistical definitions (HR, CI etc.) for each set of parentheses.

* The information on page 5 ('what's known' and 'what does this study add') is not necessary. Please remove.

* Thank you for including the STROBE checklist. Please amend to use section and paragraph numbers, rather than page numbers. Please also provide this file as a separate supplement, rather than as part of the supplementary materials.

FUNDING STATEMENT

* Thank you for including your funding statement. We note that this is very long, and seems duplicated in the metadata. Please consider whether all these funders indeed provided support for this specific study. Please amend and make more concise if possible.

AUTHOR SUMMARY:

* Under 'why was this research done' please consider adding an additional point, to provide more background to a layman's' audience.

* Under 'what did the researchers do and find' please provide a bit more explanation in the first bullet point. Please also consider switching point 2 and 3, to provide the data in a chronological order.

* In the author summary, in the final bullet point of 'What Do These Findings Mean?', please include the main limitations of the study in non-technical language.

DATA AVAILABILITY

* PLOS Medicine requires that the de-identified data underlying the specific results in a published article be made available, without restrictions on access, in a public repository or as Supporting Information at the time of article publication, provided it is legal and ethical to do so. Please see the policy at http://journals.plos.org/plosmedicine/s/data-availability and FAQs at http://journals.plos.org/plosmedicine/s/data-availability#loc-faqs-for-data-policy

* Please provide reasoning why the data cannot be shared, and what requirements researchers need to adhere to to obtain access to the data fro the Finnish pediatric diabetes registry and the Finndata.

* We do not accept the statement "The de-identified aggregated data that underlie the results reported in this Article can be obtained from the corresponding author (mikael.knip@helsinki.fi) upon reasonable request." Please provide this information, or provide a statement why this cannot be publicly available, and how researchers could get access to this information. Please note that the contact in that case cannot be a study author.

* Please include the statement on code availability in the data availability statement. Please see guidance here: https://journals.plos.org/plosmedicine/s/code-sharing-guidance#loc-data-availability-statement-examples

* Please note that we do require code to be made available, and this cannot be upon request. Please provide the code as a supplement, or provide a URL to where the code can be found (e.g. Github, with a Zenodo DOI).

TEXTUAL COMMENTS:

Intro:

-line 145: please remove ‘a series of research articles. This either needs references, or more detail, as you provide in the following sentences.

-line 152: include ‘incidence’. Also consider adding more detail, e.g something like: “it is unknown whether type 1 diabetes incidence after the pandemic returned to pre-pandemic levels”.

Methods:

225: please consider adding a definition for ‘strict’ and ‘loose’.

FIGURES

* Please consider avoiding the use of red and green in order to make your figure more accessible. This also applies to supplementary figures.

* Figure 1: please clarify what the blue and white colours of the bars signify are in the legend.

* Figure 2: please clarify what the colours used in the figure mean in the legend.

Comments from Reviewers:

Reviewer #2: In this final resubmission, the authors discuss prior critiques clarifying the study features and claimed originalty / impact and made some amendment to the text.

Two remaining limitations should be made more explicit.

i) The analyses compare predefined calendar periods but do not model the underlying secular trend in childhood T1D incidence. Therefore, the similarity between post-lockdown and pre-pandemic incidence does not necessarily establish a return to the expected trajectory and cannot fully distinguish a transient increase from temporal displacement/compensation superimposed on the background trend.

ii) The proposed indirect mechanisms, including reduced exposure to other infections, should be more clearly framed as speculative.

Any attachments provided with reviews can be seen via the following link:

[LINK]

# Author comment: Author response to Decision Letter 4

**DOI:** 10.1371/journal.pmed.1005288.r007

29 Sep 2026

Attachment Submitted filename: ST_Response_25.9.26.docx

# Editor report: Decision Letter 4

**DOI:** 10.1371/journal.pmed.1005288.r008

**Authors:** Suzanne De Bruijn

8 Oct 2026

Dear Dr Tall,

On behalf of my colleagues and the Academic Editor, Weiping Jia, I am pleased to inform you that we have agreed to publish your manuscript "Trends in the Incidence of Childhood Type 1 Diabetes After the COVID-19 Lockdown in Finland: An Observational Study" (PMEDICINE-D-26-00821R4) in PLOS Medicine.

Before your manuscript can be formally accepted, we have three remaining requests:

1) Please add the URL for the Findata permit application portal to your data availability statement.

2) Thank you for including links to your code. However, it seems that the Github link is missing; instead, the Zenodo link is provided twice. Please check and amend if necessary.

3) When a p value is given, please specify the statistical test used to determine it in the legend. This is specifically the case for table 2, as well as table S2 and S3.

Furthermore, you will need to complete some formatting changes, which you will receive in a follow up email. Please be aware that it may take several days for you to receive this email; during this time no action is required by you. Once you have received these formatting requests, please note that your manuscript will not be scheduled for publication until you have made the required changes.

In the meantime, please log into Editorial Manager at http://www.editorialmanager.com/pmedicine/, click the "Update My Information" link at the top of the page, and update your user information to ensure an efficient production process.

PRESS

We frequently collaborate with press offices. If your institution or institutions have a press office, please notify them about your upcoming paper at this point, to enable them to help maximise its impact. If the press office is planning to promote your findings, we would be grateful if they could coordinate with medicinepress@plos.org. If you have not yet opted out of the early version process, we ask that you notify us immediately of any press plans so that we may do so on your behalf.

We also ask that you take this opportunity to read our Embargo Policy regarding the discussion, promotion and media coverage of work that is yet to be published by PLOS. As your manuscript is not yet published, it is bound by the conditions of our Embargo Policy. Please be aware that this policy is in place both to ensure that any press coverage of your article is fully substantiated and to provide a direct link between such coverage and the published work. For full details of our Embargo Policy, please visit http://www.plos.org/about/media-inquiries/embargo-policy/.

Thank you again for submitting to PLOS Medicine. We look forward to publishing your paper.

Sincerely,

Suzanne De Bruijn, PhD

Associate Editor

PLOS Medicine
